# Relative Sense of Belonging and the Academic Achievement of Chinese Adolescents

**DOI:** 10.1007/s10964-025-02287-5

**Published:** 2025-11-13

**Authors:** Yushan Zhao, Kevin A. Gee, Caleb W. Yuan

**Affiliations:** 1 Department of Human Ecology, University of California, Davis, USA; 2 School of Education, University of California, Davis, USA; 3 Department of Educational Psychology, University of Arizona, Tucson, USA

**Keywords:** Sense of belonging, Relative sense of belonging, Chinese adolescents, Hierarchical linear modeling, Program for international student assessment (PISA)

## Abstract

For Chinese adolescents, a stronger sense of school belonging has been linked to higher academic achievement. Yet, sense of belonging has typically been analyzed only as an individual-level construct, even though its effects may depend on how their sense of belonging stands relative to their peers. This study introduces the concept of *relative sense of belonging*—how a student’s sense of belonging compares to the average belonging level in their school—and examines its association with adolescents’ academic performance, overall and by gender subgroups. The sample consisted of Chinese adolescents who participated in the 2018 Program for International Student Assessment (*N* = 12,058, *M*_age_= 15.8; *SD* = 0.30, 48% female). Results based on two-level hierarchical linear models show that higher relative sense of belonging significantly predicted higher achievement across a broad range of academic domains—math, reading, and science—for students, overall, and in reading and science for boys. However, these positive associations were not observed among girls, suggesting possible gender differences in how relative sense of belonging relates to academic outcomes. By focusing on relative sense of belonging, this study highlights that students’ standing compared with their peers’ sense of belonging can play an important role in academic achievement.

**Supplementary Information:**

The online version contains supplementary material available at 10.1007/s10964-025-02287-5.

## Introduction

Students with a stronger sense of belonging—defined as students’ need to feel accepted and supported within educational environments (Korpershoek et al., 2020)—are more likely to have better mental health (e.g., lower depressive symptoms) and better academic outcomes (e.g., higher grades, higher retention) (Pedler et al., 2022). Despite these advances, prior research typically conceptualizes belonging as an individual-level attribute, focusing on students’ personal perceptions of sense of belonging at school. One area overlooked in the current research base on school belonging is the concept of *relative sense of belonging*, which captures how a student’s own level of belonging compares to their peers’ sense of belonging within their school. Investigating relative sense of belonging is important as it may yield new theoretical and empirical insights into adolescents’ academic adjustment and well-being, especially during the developmental period when peer norms and group identity are highly salient (Meeus, 2011). In addition to the limited attention on relative sense of belonging, research has seldom explored sense of belonging in non-Western educational contexts, especially among secondary school students in China (Allen et al., 2018). Given these gaps, the present study examines the association between relative sense of belonging and academic achievement, overall and within gender subgroups using a large-scale sample of Chinese adolescents from the 2018 Program for International Student Assessment (PISA).

### Three Theoretical Frameworks Explaining Relative Sense of Belonging: The Belonging Hypothesis, Self-Determination Theory, and Social Comparison Theory

Relative sense of belonging is grounded in three theoretical frameworks: the belonging hypothesis, self-determination theory, and social comparison theory. Individuals have a fundamental need to build connections with the environment and the people around them. According to the belongingness hypothesis, the feeling of belonging is a key factor for individuals to function (Korpershoek et al., 2020). In the school setting, a student needs three basic needs to be satisfied to succeed: first is the need to feel a sense of belonging to school; second is the perception that education is useful for their future; and third is school engagement, in which students actively participate in school activities and engage in academic tasks. Researchers have argued that the first need—feeling a sense of school belonging—is a prerequisite for students to be able to fulfill the remaining needs (Smerdon, 2002). That is, only when students feel a sense of belonging to their school will they perceive their education as valuable and be motivated to put effort into it.

Additionally, self-determination theory emphasizes the importance of feeling connected to the environment, but from a slightly different perspective (Korpershoek et al., 2020). According to this theory, for students to achieve academic success, they need to feel connected to their surroundings, experience a sense of autonomy, and feel confident in their ability to complete academic tasks (Ryan & Deci, 2000). While the belongingness hypothesis views belonging as a prerequisite for student success, self-determination theory suggests that a sense of belonging functions as a mediator, helping explain why students in certain school environments achieve better academic outcomes. Specifically, students in schools that foster a stronger sense of belonging are more likely to perform better on exams than students in schools where this sense is weaker. This is because, according to self-determination theory, students are more likely to internalize information—such as academic knowledge—and foster intrinsic motivation when they are in a place where they feel they belong (Ryan & Deci, 2000).

While both the belongingness hypothesis and self-determination theory have been used interchangeably in past research to explain why a sense of belonging matters for student success, these theories have only focused on the absolute terms of the sense of belonging. Social comparison theory, instead, introduces an additional theoretical layer that can help to explain the relative sense of belonging experience. According to social comparison theory, the characteristic of schools evaluating students based on academic performance makes school a context where social comparison easily happens. Individuals tend to compare themselves with the people around them to get a sense of what their relative status is (Dijkstra et al., 2008). In schools, students compare themselves with their peers across a range of factors, including their academic achievement, physical appearance, and even personal mood (Dijkstra et al., 2008). Students may compare their own sense of belonging with that of their peers. For example, if a student perceives that classmates feel more connected to the classroom or have more friends, this contrast may stand out as a signal of their own lower sense of belonging. Past studies have assessed individuals’ social comparison processes using different methods. One approach is to present participants with a prompt and ask them to imagine comparing themselves with others in that context. For example, prompts were provided to guide mothers to imagine comparing their parenting practices with those of other mothers in an online group (Amaro et al., 2019). Another approach is to ask participants to self-report how frequently they compare themselves with others (Kuo & Yang, 2019). Those studies rely on direct measures of relative feeling. However, such measures—for example, directly asking participants how they perceive their sense of belonging relative to their peers (e.g., “Do you feel a greater sense of belonging to the school than your classmates?”)—are typically not captured in large-scale assessments, such as the PISA. Rather, more indirect measures need to be constructed that can serve as a proxy for students’ relative sense of belonging, including capturing their standing relative to their school average.

One line of social comparison research proposes the related-attributes hypothesis, which suggests that individuals are more likely to compare themselves with people similar to them. For example, students are more likely to compare themselves with same-gender peers rather than opposite-gender peers (Dumas et al., 2005). This may be because students assume that shared characteristics, such as gender, are linked to academic achievement, leading them to compare themselves with peers of the same gender. It may also be because students are more familiar with those who share similar characteristics, making them more likely to use these more similar peers as a basis for comparison. Thus, beyond examining overall relative sense of belonging, it is also important to consider students’ sense of belonging relative to their same-gender peers.

### Relative Sense of Belonging Defined

Relative sense of belonging is defined as one’s own sense of belonging relative to the average sense of belonging of their surrounding peers in school. This idea is informed by the assertion that human belonging can be attained through multiple pathways (Hirsch & Clark, 2019). According to this assertion, a sense of belonging is a multifaceted construct which can occur through several pathways: social connectedness (i.e., the need to feel connected to people around them), status-based validation (i.e., feeling accepted by gaining higher status than those around them), belonging to a community (i.e., the need to be part of a group), and light social connection (i.e., small positive interactions with people around them) (Hirsch & Clark, 2019). While the social connectedness pathway requires individuals to gain acceptance and form secure connections with those around them, the status-based pathway requires individuals to proactively self-evaluate themselves through social-comparison (Hirsch & Clark, 2019). The idea of relative sense of belonging—where individuals assess not only their own sense of belonging at school but also how it compares to that of their peers—reflects the status-based validation pathway. This pathway suggests that some individuals feel a stronger connection to their environment when they see themselves as excelling or standing out compared to others. Relative sense of belonging captures this comparison-based process. For example, a student may feel more connected not just because they feel accepted, but because they believe they belong more than their classmates—reinforcing their sense of value or standing within the school environment. Research on school belonging has mainly focused on the social connectedness (e.g., “feel comfortable with peers and classmates”) and belonging-to-a-community pathways (e.g., “feel fit in”, Fink et al., 2020). Little to no emphasis has been placed on the status-validation pathway in an attempt to understand students’ sense of belonging relative to those around them.

Notably, the measure of relative sense of belonging in the current study does not capture relative feeling; rather, it reflects students’ relative standing in their sense of belonging. This is because a direct measure of relative feeling (e.g., asking students to report how their sense of belonging compares to that of their peers) is not available in the current dataset. Nevertheless, it remains valuable to evaluate the relative sense of belonging by subtracting the average school belonging score from each student’s individual belonging score. Even though it is not a direct measure of relative feeling, it still provides insight into students’ relative standing within the school context. Belonging is inherently a social construct, and knowing whether a student’s belonging is above or below the school average reflects their position in the belonging hierarchy. This approach enables researchers to examine whether being above or below the school average in belonging is associated with important outcomes (e.g., achievement, motivation, well-being). It operationalizes the idea that students’ own sense of belonging exists relative to the broader peer context, even if not measured directly through self-reported comparison.

### The Importance of Relative Sense of Belonging

#### Relative Sense of Belonging in Adolescence

The social comparison aspect of relative sense of belonging is especially relevant during adolescence—a time when social comparison becomes a dominant strategy for self-evaluation (Krayer et al., 2008). By consciously observing peers who share similar characteristics, such as gender or ethnicity, adolescents deepen their self-awareness and engage in identity exploration (Meeus, 2011). In school settings, these comparisons often involve not just academic performance or appearance, but also social visibility and popularity. When adolescents consider who “belongs” more in school, they often look to peers with higher social visibility or popularity, implicitly linking belonging to social status. Empirical evidence supports this connection: students with higher perceived peer status report stronger feelings of belonging (Zhang et al., 2024). This suggests that adolescents’ sense of belonging is not formed in isolation but shaped in relation to how they perceive their status related to their peers.

#### Relative Sense of Belonging and Academic Achievement

Students who feel a sense of belonging in their learning environments are more likely to succeed in school. This finding has been consistently replicated across multiple academic domains—including math (Ho et al., 2005), language (Tan et al., 2022), computer science, and science (Fong et al., 2024)—and among students of various age groups, from elementary and secondary education to college. When students feel connected to their learning environments, they are more likely to engage with lectures and peers, feel motivated to learn, and ultimately benefit academically from this active engagement and motivation (Fong et al., 2024). While the positive association between sense of belonging and academic achievement is well established, it remains unclear whether relative sense of belonging also plays a similar role across multiple academic domains. Relative sense of belonging may also influence academic achievement, particularly in competitive learning environments. For example, in a less competitive classroom, students may not compare themselves with their peers very often (Stapel & Koomen, 2005), potentially resulting in minimal variation in their relative sense of belonging and reducing its relevance to academic outcomes. In contrast, in environments that emphasize social comparison, relative sense of belonging may become more salient. Students may unconsciously compare how much they feel they belong to how much their peers seem to belong (Stapel & Koomen, 2005). Through this process, relative sense of belonging becomes tied to their perceived social standing within the classroom (Zhang et al., 2024).

#### Gender Patterns in Relative Sense of Belonging

Additionally, the concept of relative sense of belonging may reveal underlying gender dynamics within belonging research. Many studies evaluate how gender affects general belonging but the findings are often mixed—while some studies suggest that girls report higher levels of school belonging (Allen et al., 2018), others find that boys feel more connected to their schools (Palikara et al., 2025). An analysis of relative sense of belonging, however, may tell a more consistent story. Specifically, gender differences might be able to find in adolescents belonging experience via a relational context.

Relative sense of belonging may matter more for boys than for girls because boys tend to be more responsive to peer status—a construct that is closely linked to belonging experiences in school settings (Zhang et al., 2024). When choosing friends, boys are more likely than girls to prioritize peers who are perceived as popular or high in social status (Shin, 2017), suggesting that peer status serves as a key reference point in how they navigate relationships. In academic settings, perceiving oneself as having higher belonging than peers may also signal elevated social standing. Indeed, in Chinese schools, students’ perceived belonging is strongly associated with their perceived peer status (Zhang et al., 2024). Taken together, this suggests that for boys, relative sense of belonging may reflect not only social integration but also a sense of prestige within the peer group—making it a more salient and psychologically loaded construct for boys than girls. Those with a stronger sense of belonging relative to their peers may serve as a source of motivation and as a result, contribute to greater academic engagement.

### Sense of Belonging and Relative Sense of Belonging among Chinese Adolescents

Chinese adolescents feel less belonging to their school compared to students from other countries. Adolescents from Hong Kong and Macau scored the lowest in their belonging experience in the 31 countries in PISA 2003 (Cortina et al., 2017). A more recent study found that Chinese adolescents from mainland provinces (i.e., Beijing, Shanghai, Jiangsu, and Zhejiang) ranked 56th out of 60th in their sense of belonging among 60 countries participating in PISA 2018 (Yang & Fan, 2023). Such a lower sense of belonging and connection to their learning environment is a concern, as past research has indicated that students’ disconnection from schools is negatively related to their well-being and academic success (Zhai et al., 2020).

In order to understand sense of belonging among Chinese adolescents, it is first important to understand the concept of *He* (harmony) which is grounded in Chinese Confucianism. *He* is the expectation that individuals maintain harmonious relationships with those around them, which is accomplished by engaging in prosocial behaviors, showing forgiveness, and expressing gratitude as means of fostering connection with others and their environment (Shek et al., 2013). While such cultural expectations encourage Chinese adolescents to cultivate close and harmonious relationships with others, the emphasis on *He* may also constrain their ability to express individual identities and perspectives—potentially weakening their sense of belonging by limiting opportunities for authentic connection with their environment (Wei et al., 2013).

Beyond the cultural expectation that can potentially suppress the expression of authentic selves in pursuit of *He*, this lower sense of belonging may also be due to the nature of the Chinese education system which is characterized by hyper competitiveness (Zhao, 2016). In China, adolescents around age 15—typically in 8th or 9th grade (i.e., the age of the PISA participants in current study)—are required to take a high-stakes exam known as the *Zhongkao*. This exam determines whether students continue on to high school, be redirected to vocational tracks, or drop out of school. Throughout the three years of middle school, both teachers and parents invest substantial effort to keep students academically on track in hopes that they will succeed in this highly competitive process. However, such a competitive school climate has been shown to significantly predict a lower sense of belonging (Ooi & Cortina, 2023). In a highly competitive school climate, students often experience increased stress, which can undermine their sense of belonging at school (Tholen et al., 2022).

Additionally, the competitive climate among Chinese schools may also trigger social comparison. Previous research indicates that individuals who focus on competition are more likely to recognize the differences between themselves and others—that is, they tend to engage more in social comparison (Stapel & Koomen, 2005). In contrast, individuals who focus on cooperation are more likely to notice how similar they are to others (Stapel & Koomen, 2005). In the Chinese education system, students learn in competitive environments, which may increase their tendency to compare themselves with their peers. Given that students often compare their academic performance with peers (Ye et al., 2012), they may also compare their experiences of belonging. Thus, the concept of relative sense of belonging—beyond just absolute belonging—is highly salient since it aligns closely with the competitive nature of the Chinese education system and may offer a valuable lens for understanding Chinese adolescents’ school experiences.

### Correlates of Sense of Belonging and Academic Achievement

Student- and school-level factors may influence both relative sense of belonging and academic achievement. At the student level, age and socioeconomic status (SES) are associated with achievement (Rodríguez et al., 2020; Xiao & Sun, 2021). At the school level, school SES (Perry & McConney, 2010), gender composition (proportion of girls) and school type (public vs. private; Orón Semper et al., 2021), enrollment size (Luschei & Jeong, 2021), student–teacher ratio (Zhao & Ding, 2019), average class size (Erdogdu, 2022), and school location (Zhu et al., 2018) were found to associate with student achievement. Specifically, public and private schools differ in curricula and instructional approaches, which can yield achievement differences (Orón Semper et al., 2021); gender composition may shape motivation and stereotype threat (Orón Semper et al., 2021); and smaller classes (Erdogdu, 2022) and lower student–teacher ratios (Zhao & Ding, 2019) can increase teacher access, feedback, and engagement, which in turn enhances student achievement.

## Current Study

Prior research has primarily focused on Chinese adolescents’ own sense of belonging and its effect on their academic achievement; however, how they perceive their belonging relative to their peers—*relative sense of belonging*—has yet to be fully examined. By examining relative sense of belonging among Chinese adolescents, the current study seeks to understand how students’ sense of belonging, when above or below their school’s average level of belonging, is associated with academic success. Based on belongingness theory, self-determination theory, and social comparison theory, it was hypothesized that the socially comparative nature of belonging—above and beyond one’s peers’ average belonging—plays a meaningful role in shaping academic outcomes (Hypothesis 1). Further, given boys heightened responsiveness to social comparisons, relative sense of belonging was hypothesized to be more salient to their outcomes (Hypothesis 2).

## Methods

### Dataset and Sample

This study uses cross-sectional data from the Program for International Student Assessment (PISA) 2018, sponsored by the Organization for Economic Co-operation and Development (OECD). PISA assesses student achievement in reading, mathematics and science across 79 countries and includes surveys capturing student, family and institutional level factors. PISA utilizes a two-stage stratification sampling method to gather data that is representative of the population of students around 15 years old across each country (OECD, 2022). First, schools are stratified based on various factors including region, type of school, funding, school size, district, and composition of minority students. Second, after stratification, 42 students were then selected in each school with equal probability. This study focuses on the sample of Chinese students across 361 schools from *Beijing*,* Shanghai*,* Jiangsu*,* and Zhejiang* provinces (*N* = 12,058). Students in the current study were 15.75 years old on average (*SD* = 0.30) and 48% were female.

### Measures

#### Covariates

The study includes the following covariates: age, student ESCS, school ESCS, proportion of girls in school, school size, class size, school type (private vs. public), school location (i.e., a large city), and student–teacher ratio. Specifically, average school belonging was calculated as the average of students’ sense of belonging within a given school. The student ESCS is a composite measure of parental education, parental occupation, and home environment created by PISA scholars, and it is used as an index to assess participants’ SES backgrounds. The school ESCS is the mean of the student ESCS within a given school.

#### Sense of Belonging

Sense of belonging was assessed using six items from the PISA 2018 student survey that measured students’ perceptions of their connection and acceptance within the school environment. Students were asked the extent to which they agreed (1 = *strongly agree*, 2 = *agree*, 3 = *disagree*, 4 = *strongly disagree*) with the following six statements: (1) I feel like an outsider (or left out of things) at school; (2) I make friends easily at school (reverse scored); (3) I feel like I belong at school (reverse scored); (4) I feel awkward and out of place in my school; (5) Other students seem to like me (reverse scored); (6) I feel lonely at school. Items two, three, and five were reverse coded so that higher scores reflect a stronger sense of belonging. The six items demonstrated high internal consistency reliability (α = 0.81). These items served as indicators for the latent construct of sense of belonging. Categorical confirmatory factor analysis (Categorical CFA) was used to generate factor scores based on the six items. The categorical CFA demonstrated good model fit (𝝌²(6) = 211.95, *p* < .001, CFI = 0.998, TLI = 0.996, RMSEA = 0.054, SRMR = 0.023) and the factor scores were standardized to have a mean of zero and standard deviation of one. The results of this analysis and the model fit indices are presented in Table 1.

**Table 1:** Categorical confirmatory factor analysis (CFA) results for six survey items used to measure sense of belonging

Latent and observed variables		Standardized factor loading
Friend (R)	I make friends easily at school.	0.60***
Belong (R)	I feel like I belong at school.	0.57***
Like (R)	Other students seem to like me.	0.58***
Outsider	I feel like an outsider (or left out of things) at school.	0.85***
Lonely	I feel lonely at school.	0.86***
Awkward	I feel awkward and out of place in my school.	0.76***

Internal Consistency Reliability of Items: *α = 0.81*

Goodness of fit index: 𝝌2(6) = 211.95, CFI = 0.998, TLI = 0.996, RMSEA = 0.054, SRMR = 0.023, *p* = .00. Friend, like, and belong were specified to correlate with one another to improve model fit. R = Reverse coded; CFI = Comparative Fit Index; TLI = Tucker-Lewis Index; RMSEA = Root Mean Square Error of Approximation; SRMR = Standardized Root Mean Square Residual.*p < .05. **p < .01. ***p < .001

### Relative Sense of Belonging

Relative sense of belonging was derived from the sense of belonging variable, calculated as each participant’s belonging factor score minus the average belonging score within their school. Statistically, this means that relative sense of belonging represents the within-school deviation of each student’s belonging from the school mean, while individual belonging reflects their absolute level of belonging. As such, students whose own belonging factor scores are farther above their own school average belonging have higher relative sense of belonging factor scores.

#### Relative Sense of Belonging for Girls

Relative sense of belonging for girls was calculated as each girl’s belonging factor score minus the average belonging score of girls within the same school. This variable was used in the analytic sample of girls to capture each girl’s standing in relation to her female peers.

#### Relative Sense of Belonging for Boys

Relative sense of belonging for boys was calculated as each boy’s belonging factor score minus the average belonging score of boys within the same school. This variable was used in the analytic sample of boys to capture each boy’s standing in relation to his male peers.

#### Academic Achievement

This study focuses on students’ academic achievement in three domains: math, reading, and science. Students’ scores in each domain consisted of ten plausible values derived through item response theory (IRT). IRT accounts for test properties (e.g., test difficulty) and test takers’ characteristics (e.g., reading skills), and it has been frequently used in large-scale assessment studies (Jewsbury et al., 2024). According to IRT, students’ plausible values in each academic domain are not their raw test scores. Rather, they are random draws from each student’s estimated distribution of proficiency in each domain. The missing values were imputed during the IRT process (Jewsbury et al., 2024) and thus, there were no missing values for the ten plausible values in the PISA 2018 dataset.

#### Missing Data

Patterns in missing data were assessed using the *finalfit* package in R. The results showed that participants who are girls or from lower socioeconomic backgrounds were more likely to have missing values on the sense of belonging items, indicating that the data are missing at random (MAR). Given this, multiple imputation was applied, which is an appropriate approach for handling missing data under the MAR assumption (Hughes et al., 2019). Multiple imputation was implemented using the *blimp* package in R, which allowed us to align the imputation model with the subsequent analytic models (Keller & Enders, 2017; Huang & Keller, 2025). The frequency of missing values is summarized in Table A1 in the appendix.

### Data Analysis

#### Statistical Method

Hierarchical Linear Modeling (HLM) was used to estimate the relationship between relative sense of belonging and Chinese students’ academic achievement. HLM was selected as a desired statistical method because it accounts for the correlation of students clustered in the same school, who are more likely to have similar experiences at the same school compared to students in other schools. Such similarity among students, depending on their attendance at the same school, violates a fundamental assumption in ordinary least squares (OLS) regression: observations for individuals are independent of each other. Therefore, estimating the effect of relative sense of belonging using OLS regression can yield inefficient estimates and statistically biased standard errors. Using HLM accounts for the degree of interdependence among students within schools and allows us to obtain efficient estimates and statistically unbiased standard errors (Woltman et al., 2012). To address the first research question, a two-level hierarchical linear model (students at Level 1 and schools at Level 2) was fitted to overall sample data for the *i*th student at the *j*th school as follows:


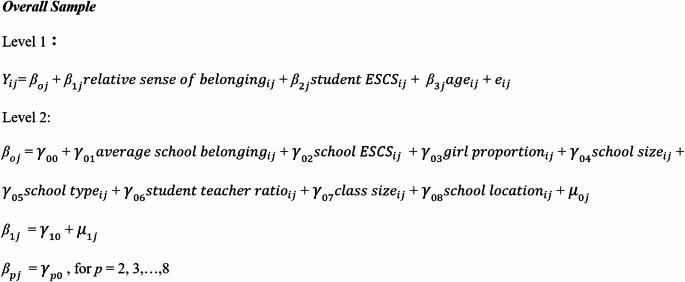


In this model, $\:{Y}_{ij}$ represents the achievement outcome of interest (math, reading, or science). The coefficient (fixed effect) of interest is $\:{\gamma\:}_{10}$ which captures how students’ relative sense of belonging (an individual student’s sense of belonging minus the average sense of belonging among all students in the same school) predicts their academic performance, net of student- and and school-level controls in the model. Based on a preliminary set of models, the slopes on relative sense of belonging at Level 1 ($\:{ꞵ}_{1j}$) was allowed to vary between schools. As is standard practice in HLM, the slopes on the control variables were fixed. For research question 2, the models were stratified by gender by first fitting the above model but using the analytic sample of boys and the relative sense of belonging measure for boys instead. This procedure was repeated for the analytic sample of girls. Equations for the boys-only and girls-only samples as well as the model building process are provided in the supplementary appendix.

Given that the present study included three analytic models—one for the full sample, one for boys, and one for girls—the *mixPV* package in R was used to fit 200 HLM models (i.e., 20 imputed datasets × 10 plausible values) to data for each of the three samples. We then pooled the results across each imputed dataset together. This modeling fitting procedure was replicated for the three academic outcome domains. The *mixPV* package is specifically designed to accommodate plausible values and incorporate sampling weights in large-scale educational datasets such as PISA (Huang, 2024). Given that this study tested multiple hypotheses (i.e., the relationship between relative sense of belonging across three different outcomes) which could lead to an inflated Type I error rate, this study adopted a Bonferroni adjusted significance level of $\:\alpha\:$ = 0.0167 with which to test the null hypothesis that there was no relationship between relative sense of belonging and the selected academic outcomes.

## Results

Table 2 presents weighted descriptive statistics based on the non-imputed datasets, both overall (*N* = 12,058 unweighted) and by gender (*n* = 6,283 for boys; *n* = 5,775 for girls). Table A2 provides the weighted descriptive statistics based on the imputed datasets. A sensitivity analysis comparing descriptive statistics from the imputed dataset (Table A2 in the Appendix) and the non-imputed dataset (Table 2) showed minimal differences in the results. Students overall scored about 591 in math, 555 in reading and 590 in science. These scores were above the OECD average by 104 points in math, 68 in reading, and 101 in science (Institute of Education Sciences, National Center for Education Statistics, 2020). For the overall sample, the mean of the average belonging factor scores is − 0.0052. Relative sense of belonging scores were standardized to have a mean of zero, thus those scores overall by each gender are 0.00. To descriptively understand how students were distributed by their individual and relative sense of belonging scores, the current study classified students into four groups using a cutoff of zero. Because both scores are standardized latent variables, a value above zero indicates above-average belonging (High), while a value of zero or below indicates average or below-average belonging (Low). Specifically, students were grouped as follows: (1) Low–Low: both individual and school-level belonging scores ≤ 0, (2) Low–High: individual score ≤ 0, school-level score > 0, (3) High–Low: individual score > 0, school-level score ≤ 0, and (4) High–High: both scores > 0.

**Table 2:** Weighted descriptive statistics for students from Beijing, Shanghai, Jiangsu, and Zhejiang participating the the 2018 program for international student assessment (PISA), overall (n (unweighted) = 12,058) and by gender (n (unweighted) = 6,283 for Boys, and n (unweighted) = 5,775 for Girls)

	Overall			Girls			Boys	
	Mean or proportion	SD		Mean or proportion	SD		Mean or proportion	SD
Predictors of academic achievement:								
Sense of belonging								
Friend (R)	2.97	0.71		2.92	0.68		3.01	0.73
Belong (R)	2.72	0.75		2.69	0.71		2.74	0.78
Like (R)	2.74	0.71		2.73	0.67		2.74	0.74
Outsider	3.01	0.74		3.01	0.70		3.01	0.77
Lonely	3.06	0.78		3.02	0.75		3.09	0.81
Awkward	3.08	0.73		3.11	0.69		3.06	0.77
Average school belonging	-0.0052	0.92		-0.0257	0.90		0.0138	0.95
Relative sense of belonging (factor score)	0.000	0.89		0.000	0.84		0.000	0.90
ESCS	-0.67	1.07		-0.65	1.05		-0.68	1.09
School ESCS	-0.67	0.67		-0.65	0.67		-0.68	0.67
Age	15.75	0.30		15.75	0.30		15.75	0.30
Girl proportion	0.48	0.08		0.49	0.08		0.47	0.08
School size	1348.17	1155.10		1372.58	1197.70		1327.51	1117.37
School type								
Public	0.88			0.89			0.89	
Private	0.12			0.11			0.11	
Student-teacher ratio	10.78	4.82		10.67	4.72		10.86	4.90
Class size	39.70	7.94		39.58	7.91		39.80	7.96
School location								
Rural area	0.12			0.12			0.12	
Small town	0.30			0.30			0.30	
Town	0.15			0.15			0.15	
City	0.220			0.20			0.20	
Large city	0.23			0.23			0.23	
Academic achievement								
Math	591.39	80.33		585.75	77.38		596.55	82.58
Science	590.45	83.19		584.15	79.83		596.21	85.74
Reading	555.24	87.23		561.89	83.93		549.15	89.70

Weighted means or proportions and standard deviations (*SD*) using non-imputed datasets. R = item reversed coded

**Fig. 1:**
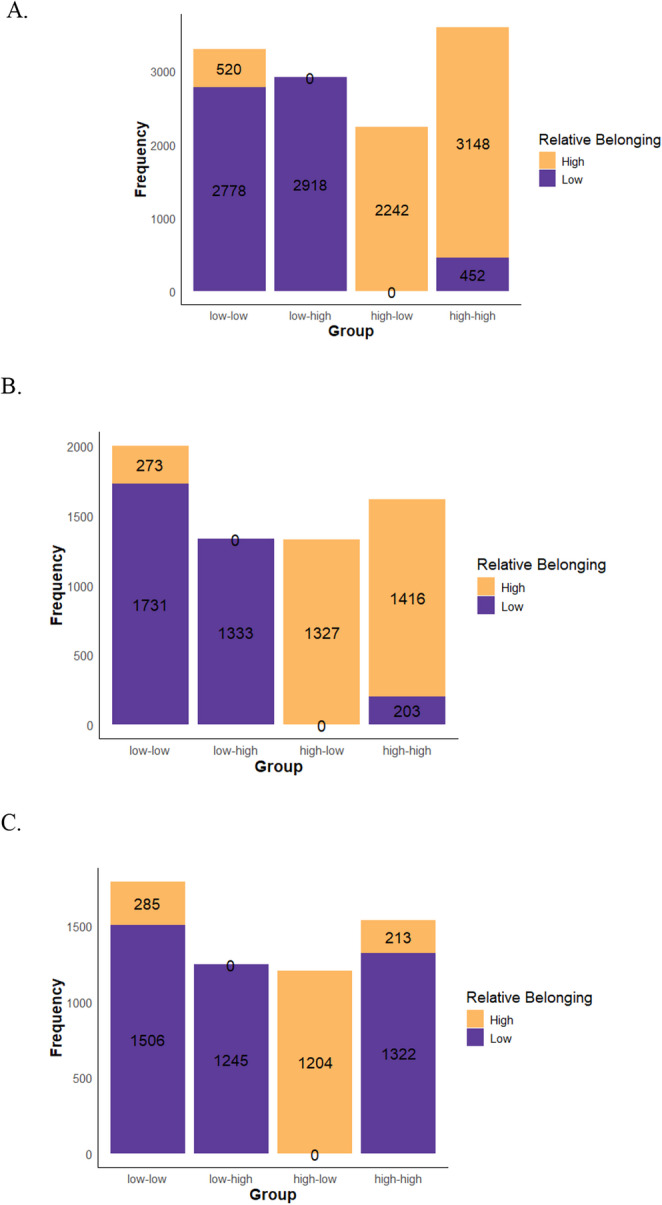
Frequency Distribution of Students by Their Individual Belonging and Relative Sense of Belonging. A Full sample. B Boys sample. C Girls sample. The labels on the x-axis (e.g., low-low) indicate students’ individual belonging scores versus their school’s average belonging score. The cutoff factor score for low versus high individual belonging was zero. The cutoff factor score for low versus high relative belonging was zero

Figure 1 displays patterns of relative sense of belonging across the full sample (Plot A), boys (Plot B), and girls (Plot C). In both the full sample and the boys’ sample, students’ individual belonging usually aligns with their relative sense of belonging. That is, students with high individual belonging were more likely to report high relative sense of belonging, regardless of their school’s average belonging, and vice versa. However, some exceptions emerged in which students’ individual belonging did not align with their relative sense of belonging. For example, a subset of students (i.e., 15%) with low individual belonging who attended schools with similarly low average belonging still reported high relative sense of belonging (see the first bar in Plot A). Additionally, while girls showed similar patterns in three of the four groups (low-low, low-high, and high-low) compared with the full and boys’ samples, the high-high group differed. More girls reported low relative sense of belonging even when both their individual belonging and the average belonging among girls in their school were high (see the third bar in plot C). In summary, these findings highlight that although one’s own individual-level of belonging often aligns with relative sense of belonging, low individual-level belonging does not always correspond with low relative sense of belonging, and vice versa. Moreover, this result suggests a gender-specific pattern in how relative sense of belonging is experienced.

### Hypothesis 1: the Association between Relative Sense of Belonging and Academic Achievement among Chinese Adolescents

Tables 3 and 4, and 5 present the fixed effects and random effects from the final HLM models for each achievement outcome (Table 3 for math, Table 4 for reading, and Table 5 for science) overall and stratified by gender. The full model-building results are presented in Tables A3 to A5 in the appendix. Based on the unconditional models (Model 1, Tables A3 for math, A6 for reading, and A9 for science in the appendix) for the full sample that includes no explanatory variables, the overall mean achievement scores were 566.70 (*SE* = 5.65) in math, 526.61 (*SE* = 6.13) in reading, and 563.88 (*SE* = 5.75) in science. The corresponding intra-cluster correlation coefficients (ICCs) were 0.45 for math, 0.46 in reading, and 0.45 in science. These ICCs indicate that 45%, 46% and 45% of the variance in math, reading, and science achievement scores, respectively, was between schools, supporting the need for HLM.

**Table 3:** Two-Level hierarchical linear models examining the relationship between relative belonging and math achievement

	Full Sample	Girls	Boys
**Fixed Effects**			
Relative sense of belonging	5.06** (1.78)	2.32 (2.37)	6.96* (2.72)
Average school belonging	13.19 (16.4)	4.99 (16.67)	19.11 (20.67)
Age	-8.39* (4.11)	-10.18* (4.92)	-7.84 (5.08)
Student ESCS	4.02** (1.23)	7.06*** (1.79)	1.42 (1.59)
School ESCS	52.7*** (8.07)	49.74*** (8.84)	57.55*** (8.59)
Proportion of girls in school	110.0** (37.0)	111.93** (37.43)	140.48*** (42.78)
School size	0.00 (0.00)	0.00 (0.00)	0.00 (0.00)
Class size	1.25** (0.47)	1.27** (0.47)	1.36** (0.53)
School type (private)	3.58 (8.81)	6.57 (9.39)	3.89 (9.30)
School location			
Small town	17.5 (12.8)	9.64 (13.31)	16.10 (15.00)
Town	20.4 (13.3)	8.90 (14.05)	26.92 (15.88)
City	-3.73 (13.1)	-16.10 (15.45)	4.81 (15.01)
Large city	-0.17 (14.4)	-5.3 (15.76)	0.19 (16.76)
Student-teacher ratio	0.73 (0.99)	0.18 (0.89)	0.91 (1.10)
Intercept	628.00*** (69.2)	659.28*** (83.51)	606.13*** (84.74)
**Random Effects**			
$\:{\sigma}_{e}^{2}$	3540.13*** (80.59)	3012.63*** (91.07)	3461.97*** (115.33)
$\:{\sigma}_{u0}^{2}$	1139.50*** (153.79)	1146.66*** (163.41)	1229.33*** (177.86)
$\:{\sigma}_{u1}^{2}$	134.37** (42.85)	254.09*** (70.80)	336.57** (108.04)

Standard errors in parentheses; the *Full Sample* model was fitted to the full dataset, the *Girls* model was fitted to the girls-only dataset, and the *Boys* model was fitted to the boys-only dataset. ESCS = PISA index of economic, social and cultural status

*p* < .10; *p < .05; **p < .01; ***p < .001

**Table 4:** Two-Level hierarchical linear models examining the relationship between relative belonging and reading achievement

	Full Sample	Girls	Boys
**Fixed Effects**			
Relative sense of belonging	6.07** (1.86)	3.10 (2.35)	8.54*** (2.55)
Average school belonging	5.18 (15.28)	-8.45 (15.33)	14.6 (20.6)
Age	-12.25** (4.09)	-10.59* (4.94)	− 0.12* (5.48)
Student ESCS	7.26*** (1.21)	9.92*** (1.56)	4.78** (1.83)
School ESCS	57.14*** (8.21)	54.93*** (8.32)	61.2*** (9.06)
Proportion of girls in school	118.74** (38.45)	99.85** (37.61)	124** (46.4)
School size	0.00 (0.00)	0.00 (0.00)	0.00 (0.00)
Class size	1.08* (0.49)	1.25** (0.42)	1.19* (0.56)
School type (private)	0.21 (9.49)	1.97 (9.35)	1.57 (9.95)
School location	9.23 (12.29)	1.32 (12.34)	11.7 (14.4)
Small town	12.99 (13.68)	5.06 (13.53)	21.4 (16.1)
Town	-7.92 (13.60)	-21.13 (16.10)	3.64 (14.8)
City	-4.06 (15.14)	-7.46 (15.37)	-0.16 (17.5)
Large city	0.73 (1.10)	0.32 (0.90)	0.79 (1.24)
Student-teacher ratio	661.45*** (67.75)	653.78*** (82.77)	644.00*** (89.3)
Intercept			
**Random Effects**	4093.63*** (63.91)	3533.57*** (86.86)	4174.10*** (75.19)
$\:{\sigma}_{e}^{2}$	1240.61*** (176.79)	1079.02*** (152.97)	1375.78*** (200.54)
$\:{\sigma}_{u0}^{2}$	187.29*** (48.20)	326.18*** (84.07)	346.40*** (82.92)
$\:{\sigma}_{u1}^{2}$	6.07** (1.86)	3.10 (2.35)	8.54*** (2.55)

Standard errors in parentheses; the *Full Sample* model was fitted to the full dataset, the *Girls* model was fitted to the girls-only dataset, and the *Boys* model was fitted to the boys-only dataset. ESCS = PISA index of economic, social and cultural status

*p* < .10; *p < .05; **p < .01; ***p < .001

**Table 5:** Two-Level hierarchical linear models examining the relationship between relative belonging and science achievement

	Full Sample	Girls	Boys
**Fixed Effects**			
Relative sense of belonging	5.95*** (1.79)	4.19 (2.53)	6.71** (2.42)
Average school belonging	5.42 (14.0)	-5.78 (13.99)	17.08 (19.76)
Age	-7.60 (4.79)	-9.49 (5.61)	-7.94 (6.06)
Student ESCS	5.10*** (1.28)	6.78*** (1.52)	3.67* (1.71)
School ESCS	56.2*** (7.49)	53.40*** (7.62)	58.98*** (8.50)
Proportion of girls in school	111** (40.9)	106.18** (39.34)	142.26** (45.02)
School size	0.00 (0.00)	0.00 (0.00)	-0.00 (0.00)
Class size	1.03* (0.47)	1.09* (0.43)	1.13* (0.55)
School type (private)	-0.18 (8.76)	3.63 (8.82)	1.29 (9.26)
School location	5.62 (11.7)	2.59 (11.98)	3.94 (13.94)
Small town	13.4 (12.8)	6.26 (12.76)	19.23 (15.04)
Town	-9.89 (13.0)	-19.14 (15.16)	-2.04 (14.34)
City	-8.13 (14.4)	-8.75 (14.49)	-7.97 (16.45)
Large city	0.94 (1.03)	0.52 (0.88)	0.96 (1.16)
Student-teacher ratio	630*** (79.0)	657.52*** (92.46)	624.36*** (98.21)
Intercept			
**Random Effects**	3762.76*** (67.18)	3177.59*** (100.17)	3771.28*** (85.84)
$\:{\sigma}_{e}^{2}$	1125.25*** (150.92)	1071.82*** (151.87)	1264.68*** (176.76)
$\:{\sigma}_{u0}^{2}$	135.22 (36.62)	294.23*** (83.05)	284.80*** (77.13)
$\:{\sigma}_{u1}^{2}$	5.95*** (1.79)	4.19 (2.53)	6.71** (2.42)

Standard errors in parentheses; the *Full Sample* model was fitted to the full dataset, the *Girls* model was fitted to the girls-only dataset, and the *Boys* model was fitted to the boys-only dataset. ESCS = PISA index of economic, social and cultural status

*p* < .10; *p < .05; **p < .01; ***p < .001

In Model 2 for each of the achievement outcomes (Tables A3 for math, A6 for reading, and A9 for science in the appendix) covariates were added including the main predictor of interest, relative sense of belonging, alongside student and school characteristics. Relative sense of belonging was significantly and positively associated with math (*b* = 3.99, *SE* = 1.14, *p* < .001, effect size = 0.05), reading (*b* = 4.17, *SE* = 1.18, *p* < .001, effect size = 0.05), and science (*b* = 4.28, *SE* = 1.38, *p* < .01, effect size = 0.05), controlling for student characteristics (i.e., age, student ESCS) and school characteristics (e.g., school ESCS, class size, school type, and location). These models included only fixed slopes for all predictors, meaning the associations between the predictors (e.g., relative sense of belonging) and outcomes (i.e., math) were assumed to be the same across all schools. In Model 3, the model was extended by allowing the slope of relative sense of belonging to vary across schools by adding a random effect for the slope on relative sense of belonging. Based on improved model fit statistics (i.e., decreases in AIC and BIC) between Models 2 and 3, the random effect for the slope was needed, suggesting that the relationship between relative sense of belonging and each achievement outcome differs across school. That is, in some schools, the link between relative sense of belonging and achievement is stronger than in others, while in others it is weaker. Based on these final model specifications, students with a higher sense of belonging relative to their peers in the same school tended to have higher scores in math, reading and science. As shown in the second column of the Table 3 for math, the coefficient for relative sense of belonging is 5.06 (*p* < .01), indicating that, after controlling for selected student and school characteristics, students whose belonging is 1 point above the average belonging for their school have a 5.06 point higher math score, an effect size (ES) of 0.06. Similarly, as Tables 4 and 5 show, a 1 point increase in relative sense of belonging above the school average belonging score is associated with a 6.07 higher reading and 5.95 higher science score, representing effect sizes of 0.07 and 0.07, respectively.

### Hypothesis 2: the Association between Relative Sense of Belonging and Academic Achievement Within Gender Subgroups

For girls, a similar model fitting procedure was followed as above (Table A4 in the appendix) and found that girls’ sense of belonging relative to other girls was not significantly associated with any of the achievement outcomes. As shown in the third column of Tables 3 and 4, and 5, although the coefficients for relative sense of belonging indicate a positive relationship with each achievement outcome, the relationship was not statistically distinguishable from zero (*p* > .05).

However, among boys, relative sense of belonging to other boys was significantly associated with their reading and science achievement. As Tables 4 and 5 show, a 1 point increase in boy’s relative sense of belonging above the school average belonging score for boys in the same school is associated with higher reading scores by 8.54 points (*p* < .001) and science scores by 6.71 points (*p* < .01), representing effect sizes of 0.10 and 0.08, respectively. As with the results for students overall, a random effect was included for the slope on relative sense of belonging that improved the model fit which suggests that the relationship between relative sense of belonging and each achievement outcome for boys varies from school to school. Although the relationship was not significant for math at the adjusted Bonferroni level (α = 0.0167), as shown in the last column of Table 3, the coefficient for relative sense of belonging is positive (*b* = 6.96) and significant at the 0.05 level.

## Discussion

Prior work has largely treated school belonging as an individual-level construct, emphasizing students’ absolute perceptions. An overlooked dimension is relative sense of belonging—the extent to which a student’s belonging is higher or lower than the school’s average sense of belonging. Little research has examined whether this peer-referenced standing is associated with academic achievement. This study addresses that gap by testing the association of relative sense of belonging and academic achievement among Chinese adolescents, and how it differs among boys and girls. First, descriptive results showed that relative sense of belonging—how a student’s belonging stands above or below the schoolwide peer average—does not always align with their absolute sense of belonging. For example, a student who reports low individual belonging and attends a school where average belonging is also low may still be classified as having high relative sense of belonging. This could be because their experiences are consistent with those of their peers, making them feel less alone. This finding underscores the importance of examining relative sense of belonging as a distinct construct. Students with higher relative sense of belonging (i.e., scores above their school’s average) had higher achievement than students with lower relative sense of belonging. In gender-stratified analyses, the same pattern held for boys: those with higher relative sense of belonging (above the boys’ school mean) showed higher reading and science achievement than those with lower relative sense of belonging. However, no significant association was found among girls suggesting that the overall results are driven, in part, by boys in the sample. Notably, the overall findings are robust across all three academic domains (math, reading, and science), suggesting that it is not a domain specific relationship. Overall, the findings suggest that students’ relative sense of belonging matters for academic performance, while also pointing to possible gender differences in its influence.

The first finding that regardless of gender, relative sense of belonging was positively associated with students’ math, reading, and science achievement builds on prior research on sense of belonging. While prior research has found that sense of belonging is positively linked to academic outcomes (Zhai et al., 2020), the findings suggest that it is not only one’s individual sense of belonging that matters, but how that belonging is situated in a relative and comparative context to one’s peers in school. Both theoretical framework and empirical evidence can help to explain this result. Social comparison theory suggests that individuals tend to evaluate themselves in relation to others, and such comparisons in school can involve academic and social-emotional experiences (Dijkstra et al., 2008). Drawing on this framework, the present study considers how students may engage in social comparison in their sense of belonging. Moreover, based on the belonging hypothesis and self-determination theories, individuals who feel a sense of belonging to their learning environment are more likely to engage with the peers and teacher, see the values of education, and develop intrinsic motivation in the study context (Korpershoek et al., 2020). When a student’s sense of belonging is higher than the school average, this relative standing may enhance their confidence, motivation, and engagement, reinforcing their academic effort and, in turn, contributing to higher academic achievement, as reflected in math, reading, and science scores in the current study.

Although no empirical studies have directly examined relative sense of belonging or its role in academic success, prior research suggests several potential mechanisms that may help explain the relationship between relative sense of belonging and student outcomes. First, feeling a higher relative sense of belonging can create a sense of psychological safety for students at school, encouraging them to participate more actively in class and persist through challenges, which are important psychological resources for student success (Gillen-O’Neel, 2021). Second, a relatively higher sense of belonging may reflect a student’s elevated social position within the school setting—particularly in the Chinese educational context, where academic competition and peer comparison are prevalent. In this context, belonging is not only about feeling accepted but may also signal perceived social status (Zhang et al., 2024). Students who experience greater relative sense of belonging may be viewed more favorably by both peers and teachers, increasing their visibility and recognition in the classroom. This heightened visibility may positively influence how students see themselves academically and could, in turn, motivate greater engagement with their schoolwork (Wei et al., 2023).

However, when the data was disaggregated by gender, different patterns emerged. Similar to the full sample, there was a positive association between relative sense of belonging and reading and science achievement among boys. This suggests that when boys’ sense of belonging exceeds the average sense of belonging of their male peers in the same school, they achieve higher in reading and science. As mentioned earlier, boys are more sensitive to social comparison (Shin, 2017). Boys, compared with girls, are more likely to choose friends and build social networks based on their level of popularity (Shin, 2017). For boys, a higher belonging compared with their male peers may serve as a signal of higher social status, which can boost their confidence and motivation, potentially increasing their school engagement and benefiting their academic success (Zhang et al., 2024). In contrast, this association was not found in the girls sample; girls’ relative sense of belonging to other girls was not significantly related to their achievement. In other words, a girl whose sense of belonging is above the within-school girls’ average does not, on average, have higher achievement. This may be due to the fact that compared with boys, girls face more systemic barriers in succeeding academically. While feeling more belonging than the same-gender peers can be a factor enhancing boys’ success in reading and science, this psychological feeling is not sufficient for girls’ success in these two academic domains. Additionally, research on popularity among Chinese adolescents suggests that the factors contributing to social recognition differ by gender (Lu et al., 2018). For girls, popularity is often tied to meeting traditional gender expectations, such as being cooperative and socially skilled, whereas boys are more likely to gain popularity through displays of intellectual ability or high socioeconomic status. Although popularity and belonging are distinct constructs, they are closely related in the Chinese context, where social comparison is common and social standing can shape how students perceive their inclusion at school (Zhang et al., 2024). These patterns suggest that the basis for relative sense of belonging may also differ by gender. Girls with higher relative sense of belonging may stand out by aligning more closely with relational and behavioral expectations, which are less directly tied to academic skills. In contrast, boys with higher relative sense of belonging may stand out due to characteristics such as intellectual ability or family background, which are more strongly linked to academic outcomes like reading and science achievement (Blums et al., 2017).

### Limitations

While the study offers new insights into relative sense of belonging, it is important to acknowledge several limitations. One limitation of this study is the absence of variables directly measuring students’ relative feelings of belonging compared to their peers. Second, although many covariates were controlled for, the cross-sectional nature of the data limits the ability to draw causal inferences because the directionality of relationships remains ambiguous. Future research should explore how relative sense of belonging develops over time, particularly whether this comparative concept becomes more salient during adolescence compared to early childhood. Additionally, while the link between relative sense of belonging and popularity was discussed as a potential mechanism underlying gender differences, the current study did not directly measure popularity or social status. Future research should include direct assessments of these constructs to more clearly examine their role in shaping relative sense of belonging and its implications for academic outcomes. Despite these limitations, the present study contributes to the literature by examining the role of relative sense of belonging in adolescents’ academic achievement, broadening the understanding of the sense of belonging experience.

### Implications

More broadly, this study’s findings suggest that efforts to support students’ sense of belonging should consider not only individual levels of belonging but also students’ standing in belonging relative to their peers. While traditional interventions focus on helping students feel accepted (Marksteiner et al., 2019), the results highlight that belonging is contextual. Students whose sense of belonging is lower than the school peer average could be at a disadvantage academically. For example, consider two students, A and B, who report the same level of belonging. If A attends a school where the average sense of belonging is high, their relative standing places them at a disadvantage compared to peers. By contrast, B’s score may position them more favorably in a school where the average sense of belonging is lower. Two students with the same belonging score may end up in very different positions depending on their peers’ average. This example underscores that belonging is not only an individual experience but also a contextual one, shaped by how students’ experiences compare to those around them. At the same time, it is important to emphasize that examining relative sense of belonging is not intended to promote competition among students. Rather, it offers a lens for understanding how school contexts may amplify or buffer the impact of belonging on academic outcomes, thereby helping educators design environments that support all students more equitably.

From a practical perspective, the findings suggest that schools should create multiple avenues for students to experience belonging rather than relying on a single dominant peer culture. When belonging is concentrated in one group or context, students who do not identify with that group may feel disadvantaged, even if their individual sense of belonging is reasonably strong. By contrast, when schools provide diverse opportunities for connection—through clubs, mentoring programs, classroom structures, and affinity groups—students are more likely to find at least one context in which they feel included. This diversification reduces the risk of being below the peer average in belonging and helps buffer the potential academic disadvantages associated with a lower relative standing. The finding that relative sense of belonging significantly predicts reading and science achievement for boys but not for girls has important implications for understanding gendered experiences in academic settings. Since relative sense of belonging is defined as the difference between a student’s own sense of belonging and the average belonging of their same-gender peers, this suggests that boys may be more sensitive to social comparisons within their peer group. In competitive educational environments, boys whose sense of belonging are above their male peers may gain greater academic confidence or engagement, which in turn supports their performance. In contrast, the non-significant effect for girls may indicate that girls’ academic outcomes are less influenced by how their belonging compares to peers, and perhaps more shaped by other factors such as SES (Jury et al., 2019) and science stereotypes (Master et al., 2021). These findings highlight the need to consider gender-specific mechanisms in how students internalize social dynamics and suggest that interventions aimed at enhancing belonging may need to be tailored differently for boys and girls.

## Conclusion

Past research has focused on individual sense of belonging, with limited attention to how students’ relative sense of belonging, defined as the difference between a student’s belonging and the school average, relates to academic achievement. This study addresses that gap by examining the role of relative sense of belonging in achievement among Chinese adolescents. The results show that relative sense of belonging is linked to achievement. In gender-stratified analyses, this positive association was observed for boys in reading and science but not for girls, suggesting that its role in academic outcomes may differ by gender. These results imply that it is important to understand adolescents’ sense of belonging through a relative lens, considering their experiences not only within the broader school environment but also in relation to the peers around them.

## Supplementary Information

Below is the link to the electronic supplementary material.

Supplementary Material 1

Supplementary Material 2

## Data Availability

This study uses data from the 2018 Programme for International Student Assessment (PISA) with the subsample of Beijing, Shanghai, Jiangsu, and Zhejiang. The 2018 PISA is available at: https://www.oecd.org/en/data/datasets/pisa-2018-database.html.

## Declarations

During the preparation of this work, the authors used Grammarly to help with grammar. After using this service, the authors reviewed and edited the content as needed and take full responsibility for the content of the publication.

## References

[CR1] Allen, K., Kern, M. L., Vella-Brodrick, D., Hattie, J., & Waters, L. (2018). What schools need to know about fostering school belonging: A meta-analysis. *Educational Psychology Review*, *30*(1), 1–34. 10.1007/s10648-016-9389-8

[CR2] Amaro, L. M., Joseph, N. T., & de los Santos, T. M. (2019). Relationships of online social comparison and parenting satisfaction among new mothers: The mediating roles of belonging and emotion. *Journal of Family Communication*, *19*(2), 144–156. 10.1080/15267431.2019.1586711

[CR3] Blums, A., Belsky, J., Grimm, K., & Chen, Z. (2017). Building links between early socioeconomic status, cognitive ability, and math and science achievement. *Journal of Cognition and Development*, *18*(1), 16–40. 10.1080/15248372.2016.1228652

[CR4] Cortina, K. S., Arel, S., & Smith-Darden, J. P. (2017). School belonging in different cultures: The effects of individualism and power distance. In *Frontiers in education*. *Frontiers Media SA*, *2*, 56. 10.3389/feduc.2017.00056

[CR5] Dijkstra, P., Kuyper, H., Van der Werf, G., Buunk, A. P., & van der Zee, Y. G. (2008). Social comparison in the classroom: A review. *Review of Educational Research*, *78*(4), 828–879. 10.3102/0034654308321210

[CR6] Dumas, F., Huguet, P., Monteil, J. M., Rastoul, C., & Nezlek, J. B. (2005). Social comparison in the classroom: Is there a tendency to compare upward in elementary school? *Current Research in Social Psychology*, *10*(12), 166–187.

[CR7] Erdogdu, F. (2022). Potential predictors of student attainment: A longitudinal study at global level. *Education and Information Technologies*, *27*(7), 9689–9711. 10.1007/s10639-022-11026-3

[CR8] Fink, A., Frey, R. F., & Solomon, E. D. (2020). Belonging in general chemistry predicts first-year undergraduates’ performance and attrition. *Chemistry Education Research and Practice*, *21*(4), 1042–1062. 10.1039/d0rp00053a

[CR9] Fong, C. J., Adelugba, S. F., Garza, M., Pinto, G. L., Gonzales, C., Zarei, P., & Rozek, C. S. (2024). A scoping review of the associations between sense of belonging and academic outcomes in postsecondary education. *Educational Psychology Review*, *36*(4), 138. 10.1007/s10648-024-09974-y

[CR10] Gillen-O’Neel, C. (2021). Sense of belonging and student engagement: A daily study of first-and continuing-generation college students. *Research in Higher Education*, *62*(1), 45–71. 10.1007/s11162-019-09570-y

[CR11] Hirsch, J. L., & Clark, M. S. (2019). Multiple paths to belonging that we should study together. *Perspectives on Psychological Science*, *14*(2), 238–255. 10.1177/1745691618803629 30517827 10.1177/1745691618803629

[CR12] Ho, E. S. C. (2005). Effect of school decentralization and school climate on student mathematics performance: The case of Hong Kong. *Educational Research for Policy and Practice*, *4*(1), 47–64.

[CR13] 10.1007/s10671-005-1546-7

[CR15] Huang, F. L. (2024). Using plausible values when fitting multilevel models with large-scale assessment data using R. *Large-scale Assessments in Education*, *12*(1), 7. 10.1186/s40536-024-00192-0

[CR14] Huang, F., & Keller, B. (2025). Working with missing data in large-scale assessments. *Large-scale Assessments in Education*, *13*(1), 13. 10.1186/s40536-025-00248-9

[CR16] Hughes, R. A., Heron, J., Sterne, J. A., & Tilling, K. (2019). Accounting for missing data in statistical analyses: Multiple imputation is not always the answer. *International Journal of Epidemiology*, *48*(4), 1294–1304. 10.1093/ije/dyz032 30879056 10.1093/ije/dyz032 PMC6693809

[CR17] Institute of Education Sciences, National Center for Education Statistics (2020). *Highlights of U.S. PISA 2018 results web report* (NCES 2020–166). U.S. Department of Education. https://nces.ed.gov/surveys/pisa/pisa2018/pdf/2020166.pdf

[CR18] Jewsbury, P. A., Jia, Y., & Gonzalez, E. J. (2024). Considerations for the use of plausible values in large-scale assessments. *Large-scale Assessments in Education*, *12*(1), 24. 10.1186/s40536-024-00213-y

[CR19] Jury, M., Aelenei, C., Chen, C., Darnon, C., & Elliot, A. J. (2019). Examining the role of perceived prestige in the link between students’ subjective socioeconomic status and sense of belonging. *Group Processes & Intergroup Relations*, *22*(3), 356–370. 10.1177/1368430219827361

[CR20] Keller, B. T., & Enders, C. K. (2017). Blimp Software Manual (Version Beta 6.7). *Los Angeles*.

[CR21] Korpershoek, H., Canrinus, E. T., Fokkens-Bruinsma, M., & De Boer, H. (2020). The relationships between school belonging and students’ motivational, social-emotional, behavioural, and academic outcomes in secondary education: A meta-analytic review. *Research Papers in Education*, *35*(6), 641–680. 10.1080/02671522.2019.1615116

[CR22] Krayer, A., Ingledew, D. K., & Iphofen, R. (2008). Social comparison and body image in adolescence: A grounded theory approach. *Health Education Research*, *23*(5), 892–903. 10.1093/her/cym076 18156148 10.1093/her/cym076

[CR23] Kuo, F. W., & Yang, S. C. (2019). In-group comparison is painful but meaningful: The moderator of classroom ethnic composition and the mediators of self-esteem and school belonging for upward comparisons. *The Journal of Social Psychology*, *159*(5), 531–545. 10.1080/00224545.2018.1515721 30199319 10.1080/00224545.2018.1515721

[CR24] Lu, T., Jin, S., Li, L., Niu, L., Chen, X., & French, D. C. (2018). Longitudinal associations between popularity and aggression in Chinese middle and high school adolescents. *Developmental Psychology*, *54*(12), 2291. 10.1037/dev0000591 30321041 10.1037/dev0000591

[CR25] Luschei, T. F., & Jeong, D. W. (2021). School governance and student achievement: Cross-national evidence from the 2015 PISA. *Educational Administration Quarterly*, *57*(3), 331–371. 10.1177/0013161x20936346

[CR26] Marksteiner, T., Janke, S., & Dickhäuser, O. (2019). Effects of a brief psychological intervention on students’ sense of belonging and educational outcomes: The role of students’ migration and educational background. *Journal of School Psychology*, *75*, 41–57. 10.1016/j.jsp.2019.06.002 31474280 10.1016/j.jsp.2019.06.002

[CR27] Master, A., Meltzoff, A. N., & Cheryan, S. (2021). Gender stereotypes about interests start early and cause gender disparities in computer science and engineering. *Proceedings of the National Academy of Sciences*, *118*(48), e2100030118. 10.1073/pnas.2100030118 10.1073/pnas.2100030118 PMC8640926 34810255

[CR28] Meeus, W. (2011). The study of adolescent identity formation 2000–2010: A review of longitudinal research. *Journal of Research on Adolescence*, *21*(1), 75–94. 10.1111/j.1532-7795.2010.00716.x

[CR29] PISA 2018 Technical Report: Overview and Methodology, PISA, OECD, OECD, & France (2022). COI: 20.500.12592/v8jmtp.

[CR30] Ooi, S. X., & Cortina, K. S. (2023). Cooperative and competitive school climate: Their impact on sense of belonging across cultures. *Frontiers in Education (Lausanne)*, *8*. 10.3389/feduc.2023.1113996

[CR31] Orón Semper, J. V., Lizasoain, I., Abaurrea, J., González-García, C., & Ayuga-Téllez, E. (2021). What kind of school organizational decisions serve to enhance sustainable personal and social growth? *Sustainability*, *13*(17), 9995. 10.3390/su13179995

[CR32] Palikara, O., Bonneville-Roussy, A., & Allen, K. A. (2025). Individual and contextual factors determining school belonging of adolescents in the UK: Evidence from PISA. *School Mental Health*, *17*(2), 598–613. 10.1007/s12310-024-09725-y

[CR33] Pedler, M. L., Willis, R., & Nieuwoudt, J. E. (2022). A sense of belonging at university: Student retention, motivation and enjoyment. *Journal of Further and Higher Education*, *46*(3), 397–408. 10.1080/0309877X.2021.1955844

[CR34] Perry, L. B., & McConney, A. (2010). Does the SES of the school matter? An examination of socioeconomic status and student achievement using PISA 2003. *Teachers College Record*, *112*(4), 1137–1162. 10.1177/016146811011200401

[CR35] Rodríguez, S., Valle, A., Gironelli, L. M., Guerrero, E., Regueiro, B., & Estévez, I. (2020). Performance and well-being of native and immigrant students. Comparative analysis based on PISA 2018. *Journal of Adolescence*, *85*, 96–105. 10.1016/j.adolescence.2020.10.001 33120032 10.1016/j.adolescence.2020.10.001

[CR36] Ryan, R. M., & Deci, E. L. (2000). Self-determination theory and the facilitation of intrinsic motivation, social development, and well-being. *The American Psychologist*, *55*(1), 68–78. 10.1037/0003-066X.55.1.68 11392867 10.1037//0003-066x.55.1.68

[CR37] Shek, D. T., Yu, L., & Fu, X. (2013). Confucian virtues and Chinese adolescent development: A conceptual review. *International Journal of Adolescent Medicine and Health*, *25*(4), 335–344. 10.1515/ijamh-2013-0031 23612532 10.1515/ijamh-2013-0031

[CR38] Shin, H. (2017). Friendship dynamics of adolescent aggression, prosocial behavior, and social status: The moderating role of gender. *Journal of Youth and Adolescence*, *46*(11), 2305–2320. 10.1007/s10964-017-0702-8 28699121 10.1007/s10964-017-0702-8

[CR39] Smerdon, B. A. (2002). Students’ perceptions of membership in their high schools. *Sociology of Education*, *75*(4), 287–305. 10.2307/3090280

[CR40] Stapel, D. A., & Koomen, W. (2005). Competition, cooperation, and the effects of others on me. *Journal of Personality and Social Psychology*, *88*(6), 1029–1038. 10.1037/0022-3514.88.6.1029 15982120 10.1037/0022-3514.88.6.1029

[CR41] Tan, Y., Fan, Z., Wei, X., & Yang, T. (2022). School belonging and reading literacy: A multilevel moderated mediation model. *Frontiers in Psychology*, *13*, 816128. 10.3389/fpsyg.2022.816128 35185734 10.3389/fpsyg.2022.816128 PMC8847447

[CR42] Tholen, R., Wouters, E., Ponnet, K., de Bruyn, S., & Van Hal, G. (2022). Academic stress, anxiety, and depression among Flemish first-year students: The mediating role of sense of belonging. *Journal of College Student Development*, *63*(2), 200–217. 10.1353/csd.2022.0015

[CR44] Wei, M., Su, J. C., Carrera, S., Lin, S. P., & Yi, F. (2013). Suppression and interpersonal harmony: A cross-cultural comparison between Chinese and European Americans. *Journal of Counseling Psychology*, *60*(4), 625–633. 10.1037/a0033413 23978268 10.1037/a0033413

[CR43] Wei, L., Jin, S., Christ, S., & French, D. C. (2023). Longitudinal associations between popularity, peer acceptance, and academic performance in adolescents. *International Journal of Behavioral Development*, *47*(6), 497–507. 10.1177/01650254231198040

[CR45] Woltman, H., Feldstain, A., MacKay, J. C., & Rocchi, M. (2012). An introduction to hierarchical linear modeling. *Tutorials in Quantitative Methods for Psychology*, *8*(1), 52–69. 10.20982/tqmp.08.1.p052

[CR46] Xiao, F., & Sun, L. (2021). Students’ motivation and affection profiles and their relation to mathematics achievement, persistence, and behaviors. *Frontiers in Psychology*, *11*, 533593. 10.3389/fpsyg.2020.533593 33519570 10.3389/fpsyg.2020.533593 PMC7841336

[CR47] Yang, W., & Fan, G. (2023). Delving into the Development of Chinese Students Based on PISA Scores. In D. Guo (Ed.), *The Frontier of Education Reform and Development in China* (pp. 107–128). Springer. 10.1007/978-981-19-6355-1_7

[CR48] Ye, Y., Mei, W., Liu, Y., & Li, X. (2012). Effect of academic comparisons on the subjective well-being of Chinese secondary school students. *Social Behavior and Personality*, *40*(8), 1233–1238. 10.2224/sbp.2012.40.8.1233

[CR49] Zhai, B., Li, D., Li, X., Liu, Y., Zhang, J., Sun, W., & Wang, Y. (2020). Perceived school climate and problematic internet use among adolescents: Mediating roles of school belonging and depressive symptoms. *Addictive Behaviors*, *110*, 106501. 10.1016/j.addbeh.2020.106501 32634681 10.1016/j.addbeh.2020.106501

[CR50] Zhang, A., Kazazi, F., Tang, K., & Howell, P. (2024). Interplay of mental state, personality, and popularity among peers in shaping belongingness of first-year students: A cross-sectional study. *PLOS Mental Health*, *1*(2), e0000019. 10.1371/journal.pmen.0000019 41661776 10.1371/journal.pmen.0000019 PMC12798505

[CR51] Zhao, X. (2016). Why and how has the Chinese discourse of competition in education rapidly changed within three decades? *Berkeley Review of Education*, *6*(1), 5. 10.5070/B86110043

[CR52] Zhao, Y., & Ding, C. (2019). The association between students mathematic knowledge and factors related to students, parents, and school: A cross-cultural comparison study. *International Journal of Educational Research*, *93*, 210–217. 10.1016/j.ijer.2018.11.006

[CR53] Zhu, Y., Kaiser, G., & Cai, J. (2018). Gender equity in mathematical achievement: The case of China. *Educational Studies in Mathematics*, *99*(3), 245–260. 10.1007/s10649-018-9846-z

