# Supplementary material for: Relative Sense of Belonging and the Academic Achievement of Chinese Adolescents
Source: J Youth Adolesc. 2025 Nov 13;55(4):918–32. doi: 10.1007/s10964-025-02287-5 (PMC13076513; doi:10.1007/s10964-025-02287-5)
Supplement: Supplementary file 1 — Supplementary Material 1 [file 10964_2025_2287_MOESM1_ESM.docx]

**Relative Sense of Belonging and the Academic Achievement of Chinese Adolescents**

**Appendix**

**Hierarchical Linear Model Equations for Girls and Boys Samples**

***Girls sample***

Level 1：

$Y_{ij}$ = $ꞵ_{oj}$+ $ꞵ_{1j}{relative sense of belonging}_{ij}$ + $ꞵ_{2j}{student ESCS}_{ij}$ + $ꞵ_{3j}{age}_{ij}$ + $e_{ij}$

Level 2:

$ꞵ_{oj}$= $\gamma_{00}$ + $\gamma_{01}{average school belonging}_{ij}$ + $\gamma_{02}{school ESCS}_{ij}$ + $\gamma_{03}{girl proportion}_{ij}$ + $\gamma_{04}{school size}_{ij}$ + $\gamma_{05}{school type}_{ij}$ + $\gamma_{06}{student teacher ratio}_{ij}$ + $\gamma_{07}{class size}_{ij}$ + $\gamma_{08}{school location}_{ij}$ + $\mu_{0j}$

$ꞵ_{1j}$ = $\gamma_{10}$ + $\mu_{1j}$

$ꞵ_{pj}$ = $\gamma_{p0}$ , for p=2,3,…,8

*Note.* Relative sense of belonging = individual girl's sense of belonging − average sense of belonging among girls in the same school.

***Boys sample***

Level 1：

$Y_{ij}$ = $ꞵ_{oj}$+ $ꞵ_{1j}{relative sense of belonging}_{ij}$ + $ꞵ_{2j}{student ESCS}_{ij}$ + $ꞵ_{3j}{age}_{ij}$ + $e_{ij}$

Level 2:

$ꞵ_{oj}$= $\gamma_{00}$ + $\gamma_{01}{average school belonging}_{ij}$ + $\gamma_{02}{school ESCS}_{ij}$ + $\gamma_{03}{girl proportion}_{ij}$ + $\gamma_{04}{school size}_{ij}$ + $\gamma_{05}{school type}_{ij}$ + $\gamma_{06}{student teacher ratio}_{ij}$ + $\gamma_{07}{class size}_{ij}$ + $\gamma_{08}{school location}_{ij}$ + $\mu_{0j}$

$ꞵ_{1j}$ = $\gamma_{10}$ + $\mu_{1j}$

$ꞵ_{pj}$ = $\gamma_{p0}$ , for p=2,3,…,8

*Note.* Relative sense of belonging = individual boy's sense of belonging − average sense of belonging among boys in the same school.

**Model Building Procedure**

Several HLM models were fit to the data to examine how student-level (level 1) and school-level (level 2) variables were associated with academic achievement. We used a bottom-up approach (Hox, 2010), starting with an unconditional model (also called the null model, step 1) and then fitting progressively more complex models (steps 2 and 3). For illustration purposes, we show our model building process for our math outcome below. We followed the same process for reading and science scores.

Model 1: Unconditional model (null model)

The first step was to fit a model without any explanatory variables:

*Math* = 566.70 (5.65) + $e_{ij}$ + $\mu_{0j}$

where $e_{ij}$ is how student *i*’s math score differs from their own school’s average math score, and $\mu_{0j}$ is how school *j*’s math score differs from the overall average math score.

This step establishes within-school variations ($\sigma_{e}^{2}$) and between-school variations ($\sigma_{u0}^{2}$). Using this information, we calculated the Intra-class Correlation Coefficient (ICC):

ICC = $\frac{Between-school variations}{Between-school variations + Within-school variations}$ = $\frac{\sigma_{u0}^{2}}{\sigma_{u0}^{2} + \sigma_{e}^{2}}$ = $\frac{3002.05}{3002.05 + 3679.66}$ = .45

This ICC value indicated that approximately 45% of the variance in math scores was due to differences between schools, while the remaining 55% reflected differences within schools. This supports the use of HLM—although within-school variation was still larger, the considerable proportion of between-school variance (nearly half of the total) signals the importance of accounting for school-level clustering.

Model 2: Random intercept model

Fixed predictors such as relative belonging, school-level covariates (e.g., school type), and student-level covariates (e.g., age) were added to the null model. This model is referred to as a random intercept model because only the intercept is allowed to vary across schools. In other words, schools are allowed to have different average math scores based on their characteristics (e.g., school type, school location). However, the relationship between relative belonging and math scores is assumed to be the same across all schools (i.e., the slope is fixed).

Model 3: Random intercept + random slope model

A random slope for relative belonging was added to the random intercept model, allowing the relationship between relative belonging and math scores to vary across schools. From Step 1 to Step 3, we sequentially built and compared three models. Based on the Bayesian Information Criterion (BIC) and Akaike Information Criterion (AIC), Model 3 in Step 3 demonstrated the best fit, yielding the lowest BIC and AIC values among all models (Muthén & Muthén, 2004). We replicated the same model-building procedure for the girls-only and boys-only subsamples. The results of this process are presented in Tables A3, A4, and A5 in this appendix.

Hox, J. J. (2010). Multilevel analysis: Techniques and applications (2nd ed.). New York: Routledge.

**Table A1**

*Missing values frequency*

|  |  |  | | Missing values | | | | | | |
| --- | --- | --- | --- | --- | --- | --- | --- | --- | --- | --- |
|  | Full sample | | | |  | Girls | |  | Boys | |
|  | n | | % | |  | n | % |  | n | % |
| Sense of belonging | | | | | | | | | | |
| Friend (R) | 105 | | .87 | |  | 52 | .90 |  | 53 | .84 |
| Belong (R) | 113 | | .94 | |  | 49 | .85 |  | 64 | 1.02 |
| Like (R) | 96 | | .80 | |  | 44 | .76 |  | 52 | .83 |
| Outsider | 90 | | .75 | |  | 44 | .76 |  | 46 | .73 |
| Lonely | 104 | | .86 | |  | 44 | .76 |  | 60 | .95 |
| Awkward | 130 | | 1.08 | |  | 56 | .97 |  | 74 | 1.18 |
| Student ESCS | 68 | | .56 | |  | 34 | .59 |  | 34 | .54 |
| School ESCS | 0 | | .00 | |  | 0 | .00 |  | 0 | .00 |
| Age | 0 | | .00 | |  | 0 | .00 |  | 0 | .00 |
| Girl proportion | 32 | | .27 | |  | 15 | .26 |  | 17 | .27 |
| School size | 32 | | .27 | |  | 15 | .26 |  | 17 | .27 |
| School type | 0 | | .00 | |  | 0 | .00 |  | 0 | .00 |
| Student-teacher ratio | 32 | | .27 | |  | 15 | .26 |  | 17 | .27 |
| Class size | 0 | | .00 | |  | 0 | 0 |  | .00 | 0 |
| School location | 0 | | .00 | |  | 0 | 0 |  | .00 | 0 |
| Math | 0 | | .00 | |  | 0 | .00 |  | 0 | .00 |
| Science | 0 | | .00 | |  | 0 | .00 |  | 0 | .00 |
| Reading | 0 | | .00 | |  | 0 | .00 |  | 0 | .00 |

*Note.* R = Reverse coded

**Table A2**

*Weighted descriptive statistics for imputed datasets (n = 200)*

|  | Overall | |  | Girls | |  | Boys | |
| --- | --- | --- | --- | --- | --- | --- | --- | --- |
|  | Mean or proportion | *SD* |  | Mean or proportion | *SD* |  | Mean or proportion | *SD* |
| Predictors of math achievement: | | | | | | | | |
| Sense of belonging |  |  |  |  |  |  |  |  |
| Friend (R) | 2.97 | .71 |  | 2.92 | .68 |  | 3.01 | .73 |
| Belong (R) | 2.71 | .75 |  | 2.69 | .71 |  | 2.73 | .78 |
| Like (R) | 2.73 | .71 |  | 2.73 | .66 |  | 2.74 | .74 |
| Outsider | 3.01 | .74 |  | 3.00 | .70 |  | 3.01 | .77 |
| Lonely | 3.06 | .78 |  | 3.02 | .75 |  | 3.09 | .81 |
| Awkward | 3.08 | .73 |  | 3.11 | .67 |  | 3.06 | .77 |
| ESCS | -.67 | 1.07 |  | -.65 | 1.05 |  | -.68 | 1.09 |
| School ESCS | -.56 | .63 |  | -.53 | .62 |  | -.58 | .64 |
| Age | 15.75 | .30 |  | 15.75 | .30 |  | 15.75 | .30 |
| Girl proportion | .48 | .08 |  | .49 | .08 |  | .47 | .08 |
| School size | 1346.52 | 1156.13 |  | 1371.07 | 1198.97 |  | 1325.80 | 1118.21 |
| School type | | | | | | | | |
| Public | .88 | |  | .89 | |  | .89 | |
| Private | .12 | |  | .11 | |  | .11 | |
| Student-teacher ratio | 10.77 | 4.82 |  | 10.67 | 4.73 |  | 10.85 | 4.91 |
| Class size | 39.70 | 7.94 |  | 39.58 | 7.91 |  | 39.80 | 7.96 |
| School location | | | | | | | | |
| Rural area | .12 | |  | .12 | |  | .12 | |
| Small town | .30 | |  | .30 | |  | .30 | |
| Town | .15 | |  | .15 | |  | .15 | |
| City | .20 | |  | .20 | |  | .20 | |
| Large city | .23 | |  | .23 | |  | .23 | |
| Math | 591.39 | 80.33 |  | 585.75 | 77.38 |  | 596.55 | 82.58 |
| Science | 590.45 | 83.19 |  | 584.15 | 79.83 |  | 596.21 | 85.74 |
| Reading | 555.24 | 87.23 |  | 561.89 | 83.93 |  | 549.15 | 89.7 |

*Note.* R = Reverse coded. Weighted means or proportions and standard deviations (*SD*). For the predictors, the imputed datasets with the math outcome were used to compute these descriptive statistics. The descriptive statistics for science and reading scores are based on the respective imputed datasets for those outcomes.

**Table A3**

*Two-level hierarchical models for math outcome for full sample*

|  | **Model 1** | **Model 2** | **Model 3** |
| --- | --- | --- | --- |
| **Fixed Effects** |  |  |  |
| Relative sense of belonging |  | 3.99***  (1.14) | 5.06**  (1.78) |
| Average school belonging |  | 15.70  (17.7) | 13.19  (16.4) |
| Age |  | -8.72*  (4.10) | -8.39*  (4.11) |
| Student ESCS |  | 3.99***  (1.21) | 4.02**  (1.23) |
| School ESCS |  | 54.10***  (8.63) | 52.7***  (8.07) |
| Girl proportion |  | 117.00**  (37.4) | 110.0**  (37.0) |
| School size |  | -.00  (.00) | .00  (.00) |
| Class size |  | 1.22*  (.51) | 1.25**  (.47) |
| School type (private) |  | 5.65  (8.77) | 3.58  (8.81) |
| School location (small town) |  | 12.2  (13.6) | 17.5  (12.8) |
| School location (town) |  | 17.2  (13.8) | 20.4  (13.3) |
| School location (city) |  | -7.20  (14.0) | -3.73  (13.1) |
| School location (large city) |  | -3.16  (15.2) | -.17  (14.4) |
| Student-teacher ratio |  | .78  (1.13) | .73  (.99) |
| Intercept | 566.70***  (5.65) | 635.00***  (65.40) | 628.00***  (69.2) |
| **Random Effects** |  |  |  |
| _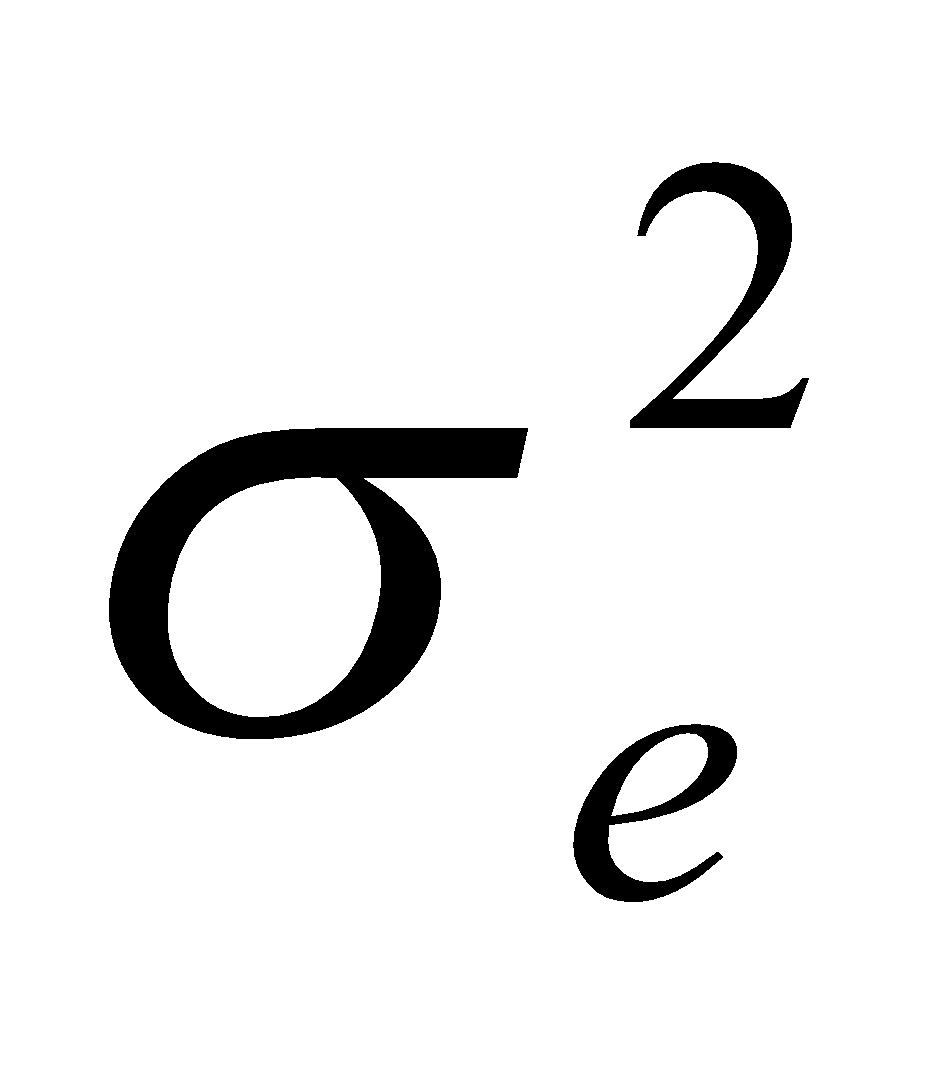_ | 3679.66***  (154.10) | 3545.38***  (148.45) | 3540.13***  (80.59) |
| _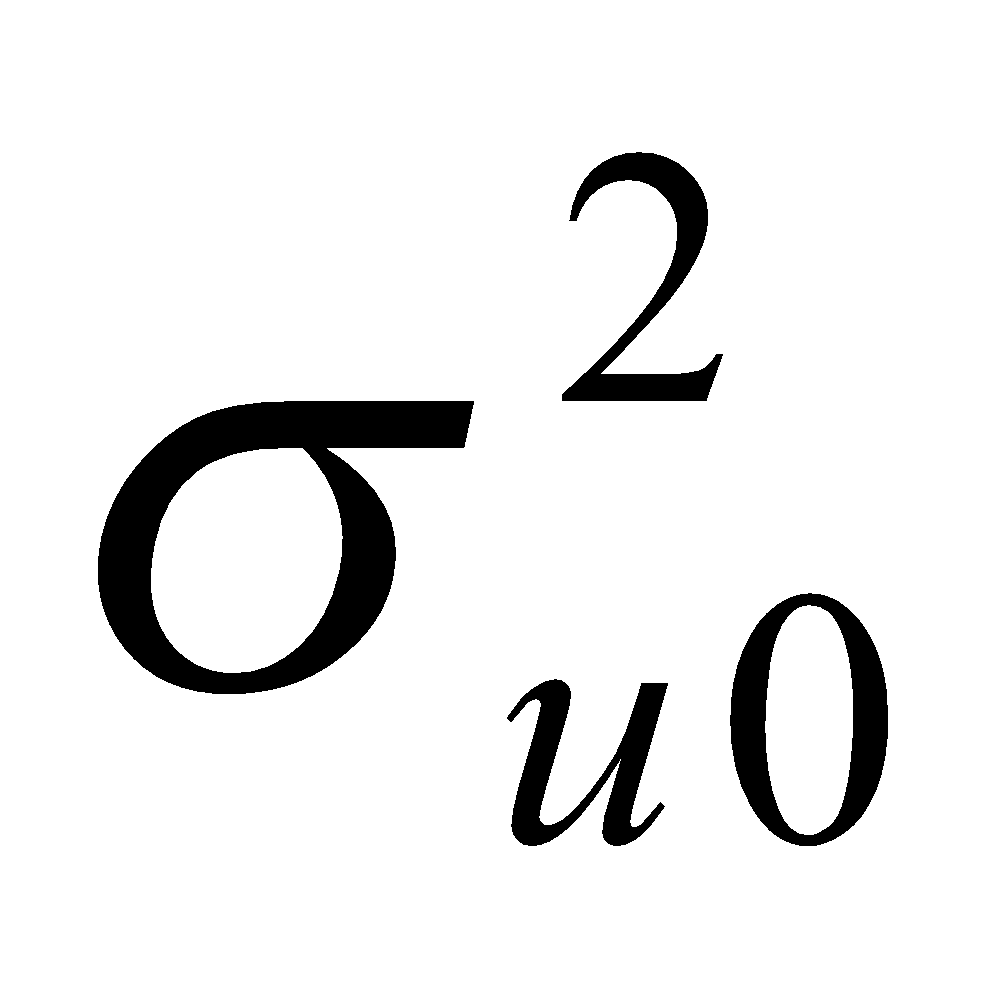_ | 3002.05***  (505.39) | 1139.57***  (151.42) | 1139.50***  (153.79) |
| _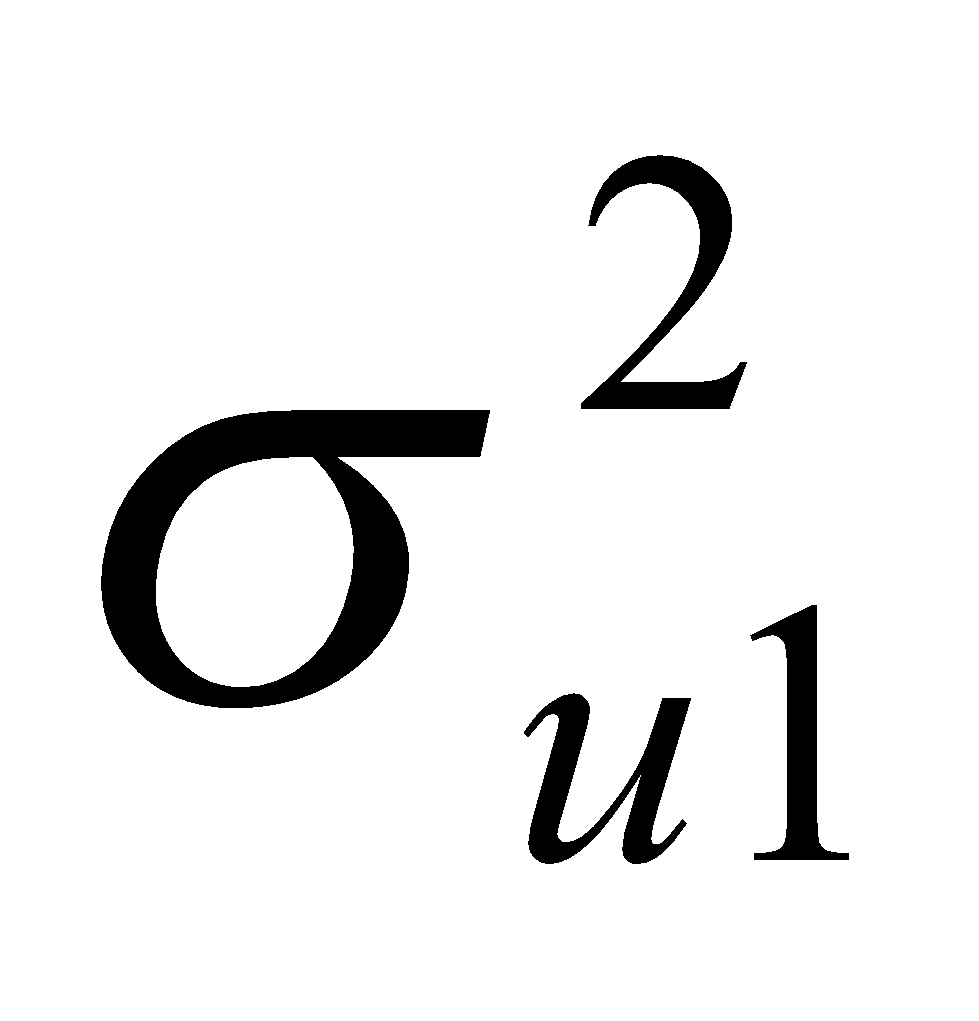_ |  |  | 134.37**  (42.85) |
| **ICC** | .45 |  |  |
| **AIC** | 10990655 | 10975550 | 10955755 |
| **BIC** | 10990677 | 10975675 | 10955895 |

*Note.* Standard errors in parentheses; Model (1) was the null model, model (2) was the model fitted with the fixed predictors, and model (3) was fitted with the fixed predictors and a random slope for relative belonging.

*p* < .10; **p* < .05; ***p* < .01; ****p* < .001

**Table A4**

*Two-level hierarchical models for math outcome for the girls-only sample*

|  | **Model 1** | **Model 2** | **Model 3** |
| --- | --- | --- | --- |
| **Fixed Effects** |  |  |  |
| Relative sense of belonging |  | 1.86  (1.55) | 2.32  (2.37) |
| Average school belonging |  | 3.09  (17.4) | 4.99  (16.67) |
| Age |  | -10.9*  (4.82) | -10.18*  (4.92) |
| Student ESCS |  | 7.09***  (1.76) | 7.06***  (1.79) |
| School ESCS |  | 49.8***  (9.11) | 49.74***  (8.84) |
| Girl proportion |  | 122**  (38.9) | 111.93**  (37.43) |
| School size |  | .00  (.00) | .00  (.00) |
| Class size |  | 1.26*  (.49) | 1.27**  (.47) |
| School type (private) |  | 4.25  (9.42) | 6.57  (9.39) |
| School location (small town) |  | 7.41  (14.1) | 9.64  (13.31) |
| School location (town) |  | 11.5  (14.6) | 8.90  (14.05) |
| School location (city) |  | -14.8  (16) | -16.10  (15.45) |
| School location (large city) |  | -4.15  (16.2) | -5.3  (15.76) |
| Student-teacher ratio |  | .31  (.94) | .18  (.89) |
| Intercept | 561.87***  (5.27) | 667***  (82.3) | 659.28***  (83.51) |
| **Random Effects** |  |  |  |
| _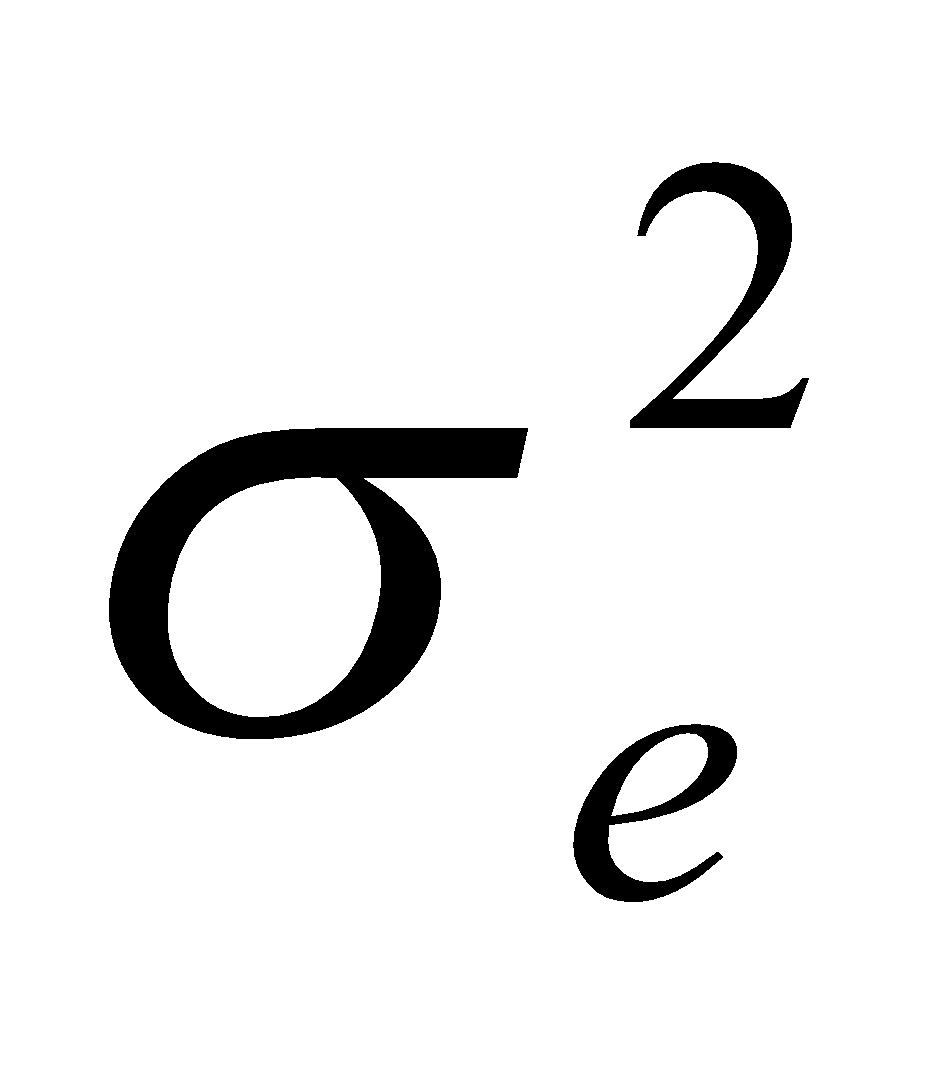_ | 3255.26***  (141.49) | 3205.68***  (132.97) | 3012.63***  (91.07) |
| _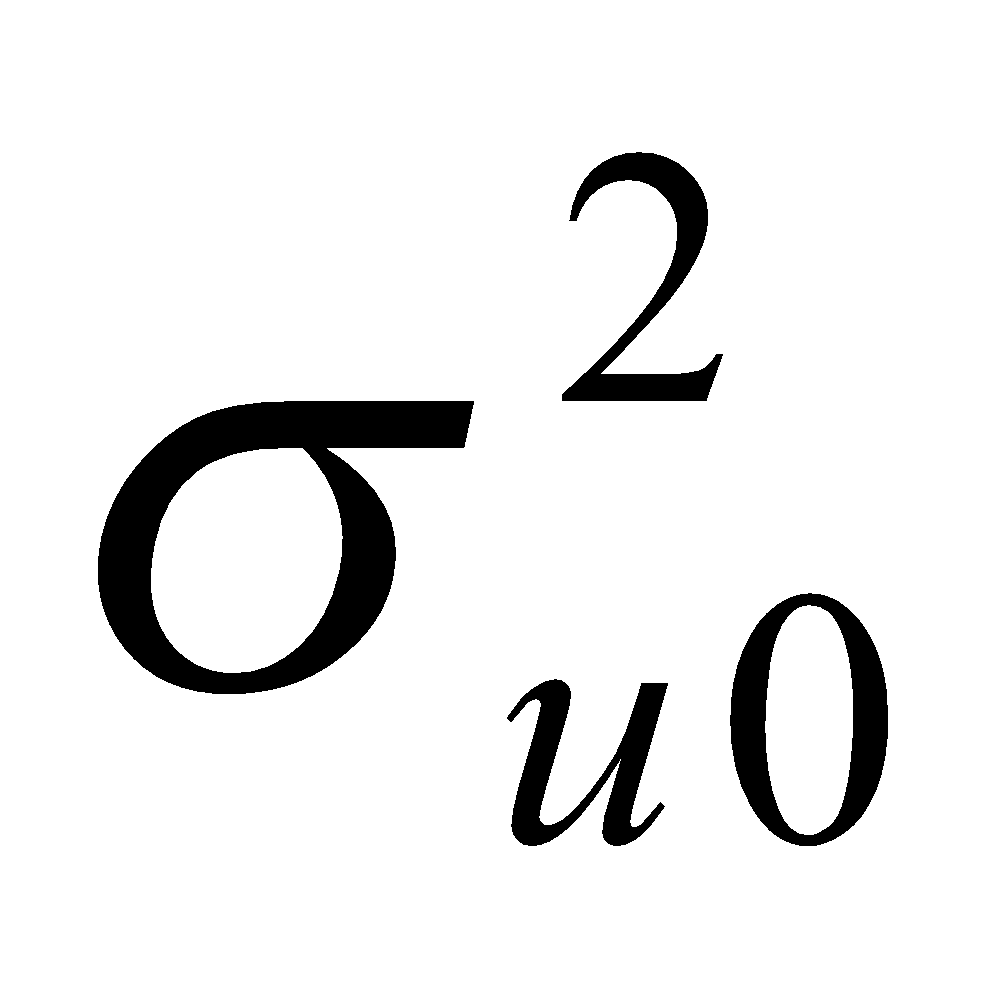_ | 2766.86***  (397.41) | 1126.12***  (153.18) | 1146.66***  (163.41) |
| _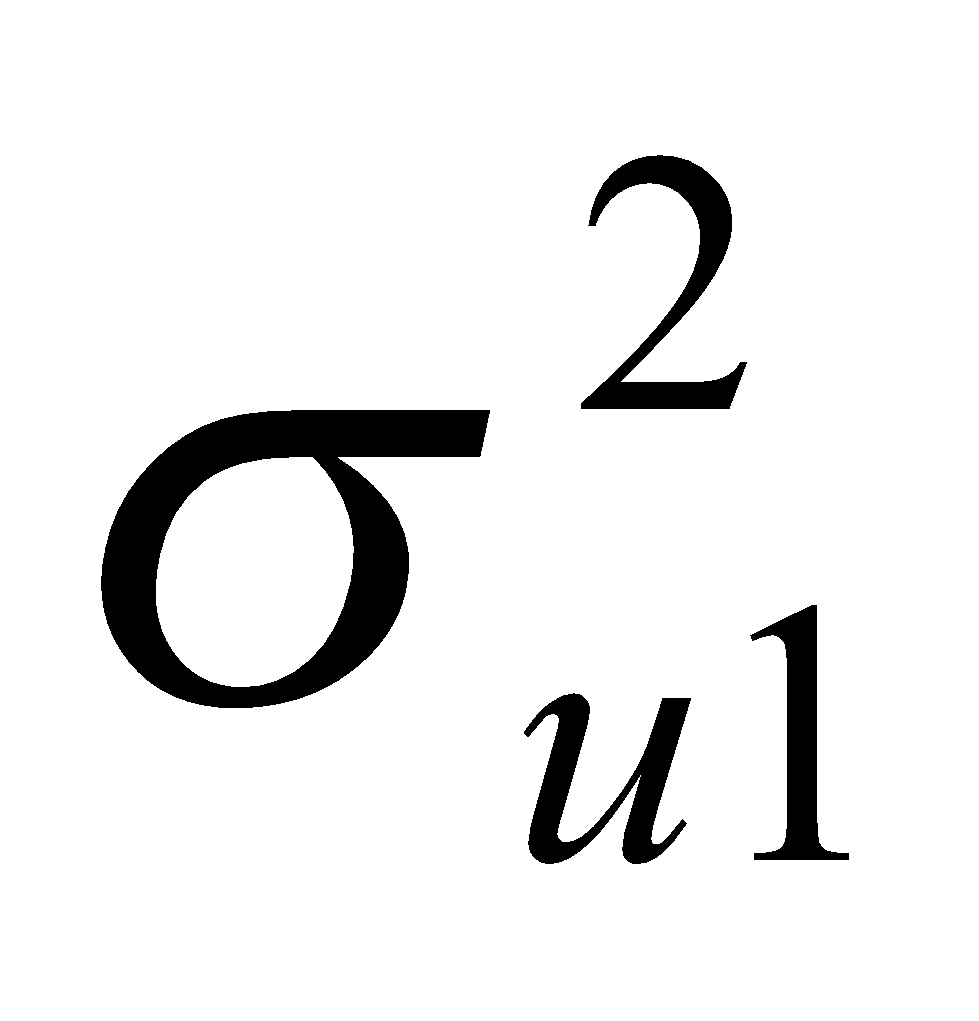_ |  |  | 254.09***  (70.80) |
| **ICC** | .46 |  |  |
| **AIC** | 5202523 | 5190092 | 5169991 |
| **BIC** | 5202543 | 5190205 | 5170118 |

*Notes:* Standard errors in parentheses; Model (1) was the null model, Model (2) was the model fitted with the fixed predictors, and Model (3) was fitted with the fixed predictors and a random slope for relative belonging.

*p* < .10; **p* < .05; ***p* < .01; ****p* < .001

**Table A5**

*Two-level hierarchical models for math outcome for boys-only sample*

|  | **Model 1** | **Model 2** | **Model 3** |
| --- | --- | --- | --- |
| **Fixed Effects** |  |  |  |
| Relative sense of belonging |  | 5.30**  (1.71) | 6.96*  (2.72) |
| Average school belonging |  | 21.9  (21.0) | 19.11  (20.67) |
| Age |  | -7.93  (4.98) | -7.84  (5.08) |
| Student ESCS |  | 1.34  (1.51) | 1.42  (1.59) |
| School ESCS |  | 58.3***  (8.94) | 57.55***  (8.59) |
| Girl proportion |  | 141***  (41.9) | 140.48***  (42.78) |
| School size |  | .00  (.00) | .00  (.00) |
| Class size |  | 1.32*  (.55) | 1.36**  (.53) |
| School type (private) |  | 6.42  (9.43) | 3.89  (9.30) |
| School location (small town) |  | 13.6  (14.8) | 16.10  (15.00) |
| School location (town) |  | 13.9  (15.6) | 26.92  (15.88) |
| School location (city) |  | 1.20  (15.1) | 4.81  (15.01) |
| School location (large city) |  | -2.08  (16.7) | .19  (16.76) |
| Student-teacher ratio |  | -8.4  (1.17) | .91  (1.10) |
| Intercept | 572.11***  (6.13) | 612***  (82.5) | 606.13***  (84.74) |
| **Random Effects** |  |  |  |
| _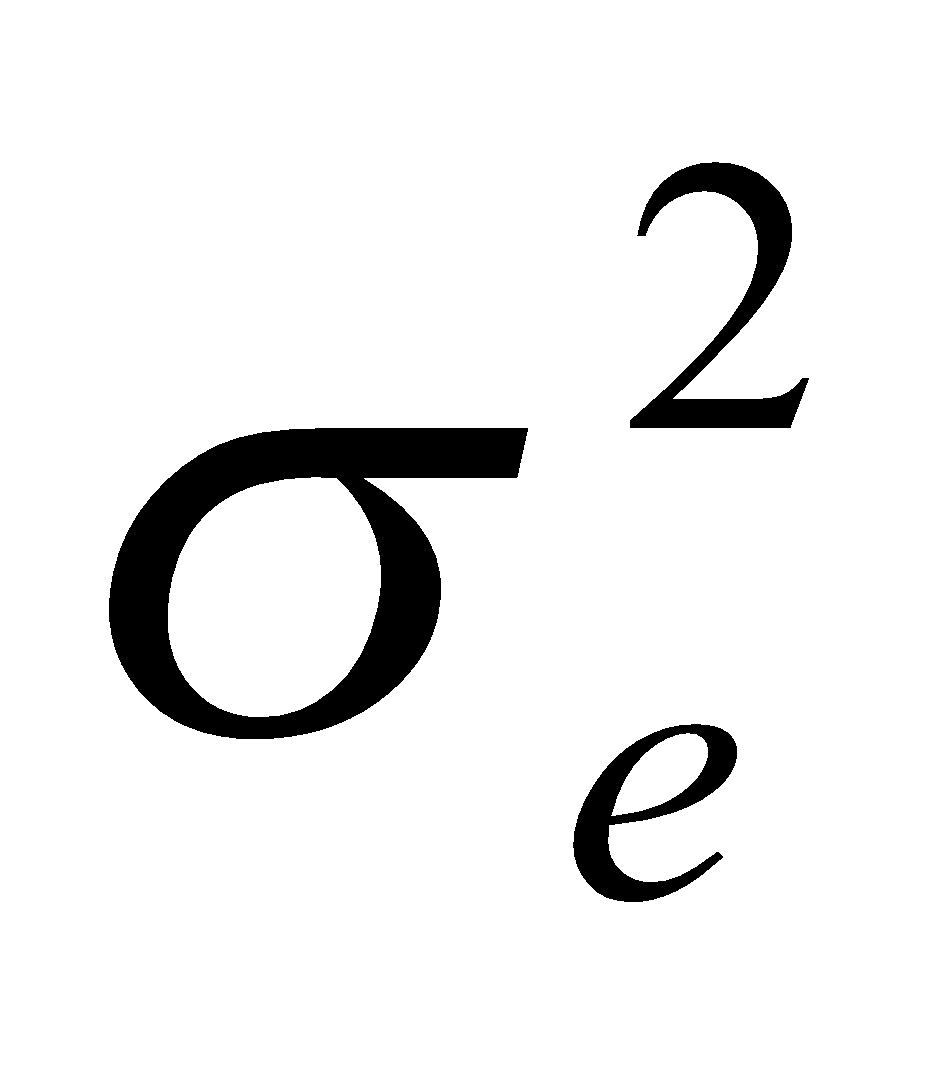_ | 3744.33***  (209.98) | 3710.80***  (203.33) | 3461.97***  (115.33) |
| _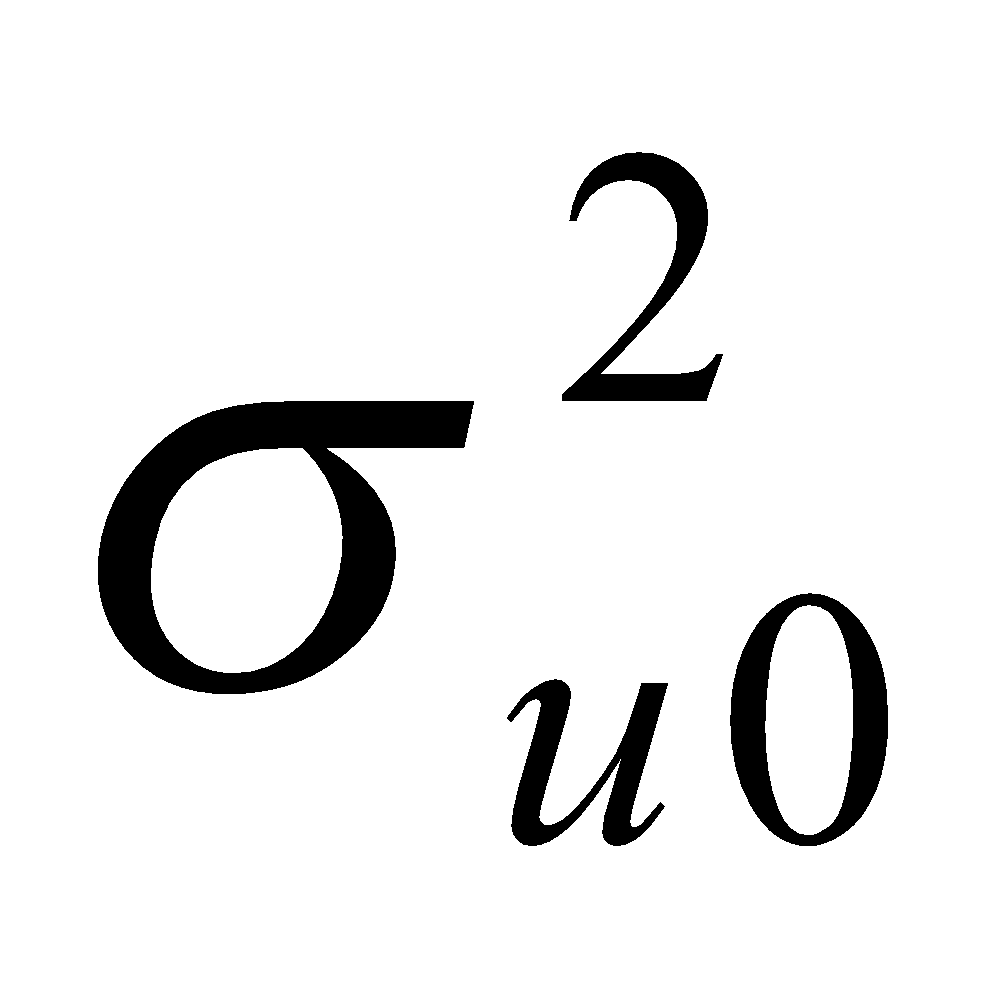_ | 3373.31***  (585.65) | 1233.90***  (181.92) | 1229.33***  (177.86) |
| _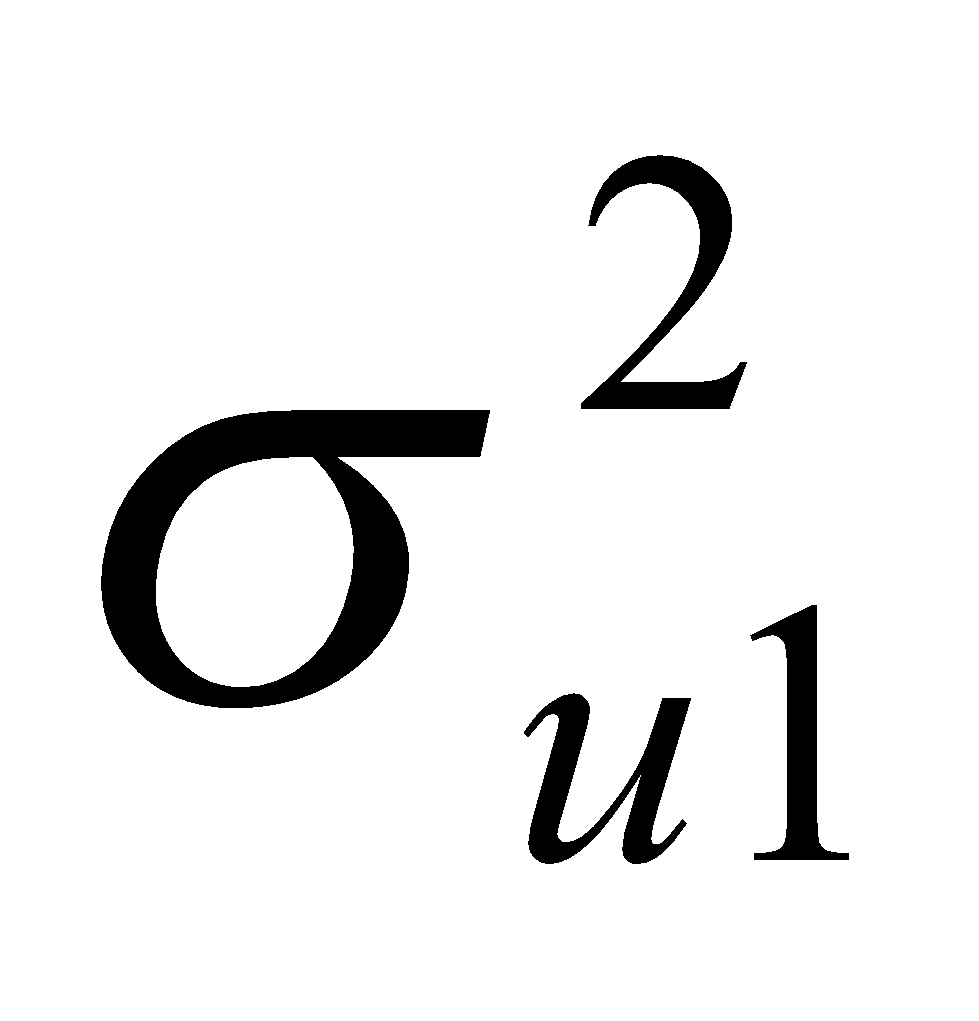_ |  |  | 336.57**  (108.04) |
| **ICC** | .47 |  |  |
| **AIC** | 5758424 | 5747843 | 5722784 |
| **BIC** | 5758444 | 5747957 | 5722912 |

*Notes:* Standard errors in parentheses; Model (1) was the null model, model (2) was the model fitted with the fixed predictors, and model (3) was fitted with the fixed predictors and a random slope for relative belonging.

*p* < .10; **p* < .05; ***p* < .01; ****p* < .001

**Table A6**

*Two-level hierarchical models for reading outcome for full sample*

|  | **Model 1** | **Model 2** | **Model 3** |
| --- | --- | --- | --- |
| **Fixed Effects** |  |  |  |
| Relative sense of belonging |  | 4.17***  (1.18) | 6.07**  (1.86) |
| Average school belonging |  | 4.39  (17.21) | 5.18  (15.28) |
| Age |  | -12.43**  (4.04) | -12.25**  (4.09) |
| Student ESCS |  | 7.29***  (1.19) | 7.26***  (1.21) |
| School ESCS |  | 58.05***  (9.11) | 57.14***  (8.21) |
| Girl proportion |  | 135.09***  (38.94) | 118.74**  (38.45) |
| School size |  | .00  (.00) | .00  (.00) |
| Class size |  | 1.05  (.54) | 1.08*  (.49) |
| School type (private) |  | 4.14  (9.54) | .21  (9.49) |
| School location (small town) |  | 4.30  (13.47) | 9.23  (12.29) |
| School location (town) |  | 9.58  (14.31) | 12.99  (13.68) |
| School location (city) |  | -10.79  (14.80) | -7.92  (13.60) |
| School location (large city) |  | -3.28  (16.26) | -4.06  (15.14) |
| Student-teacher ratio |  | .67  (1.30) | .73  (1.10) |
| Intercept | 526.61***  (6.13) | 660.68***  (68.01) | 661.45***  (67.75) |
| **Random Effects** |  |  |  |
| _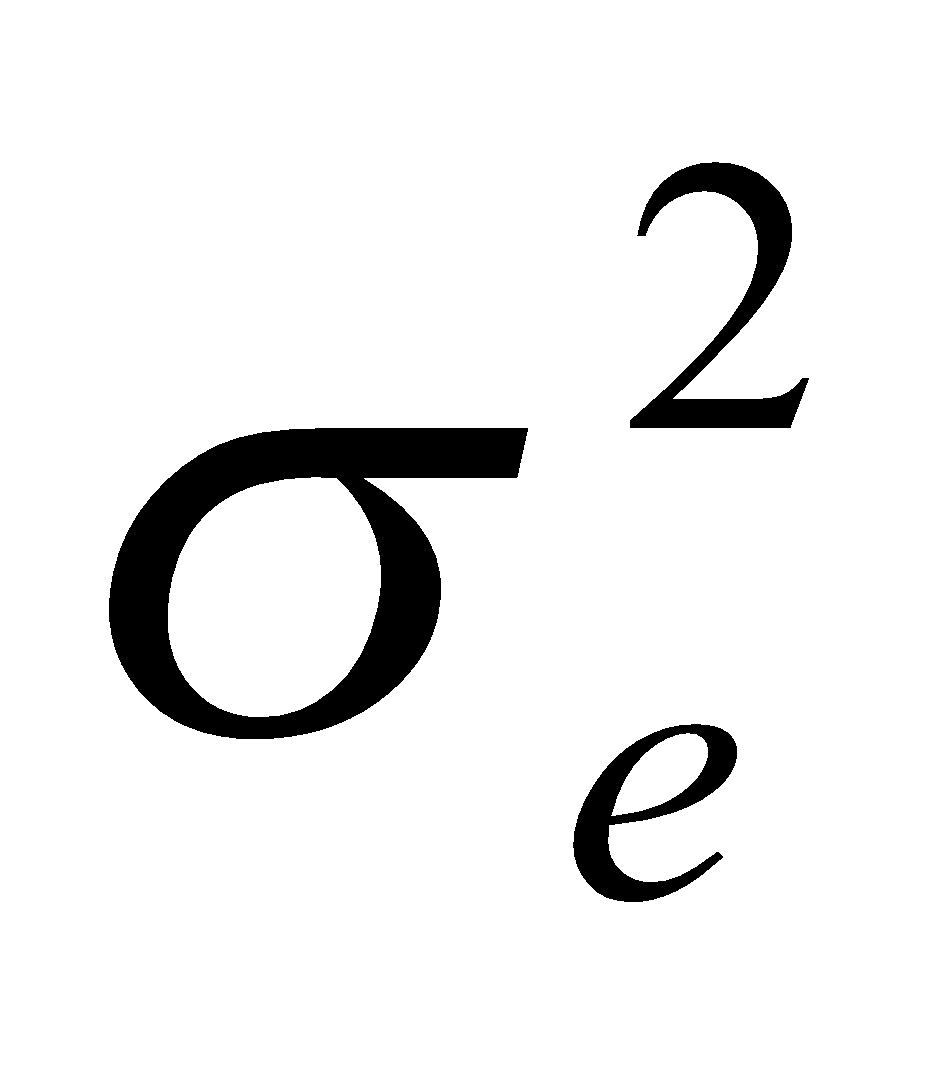_ | 4304.57***  (156.51) | 4235.56***  (149.11) | 4093.63***  (63.91) |
| _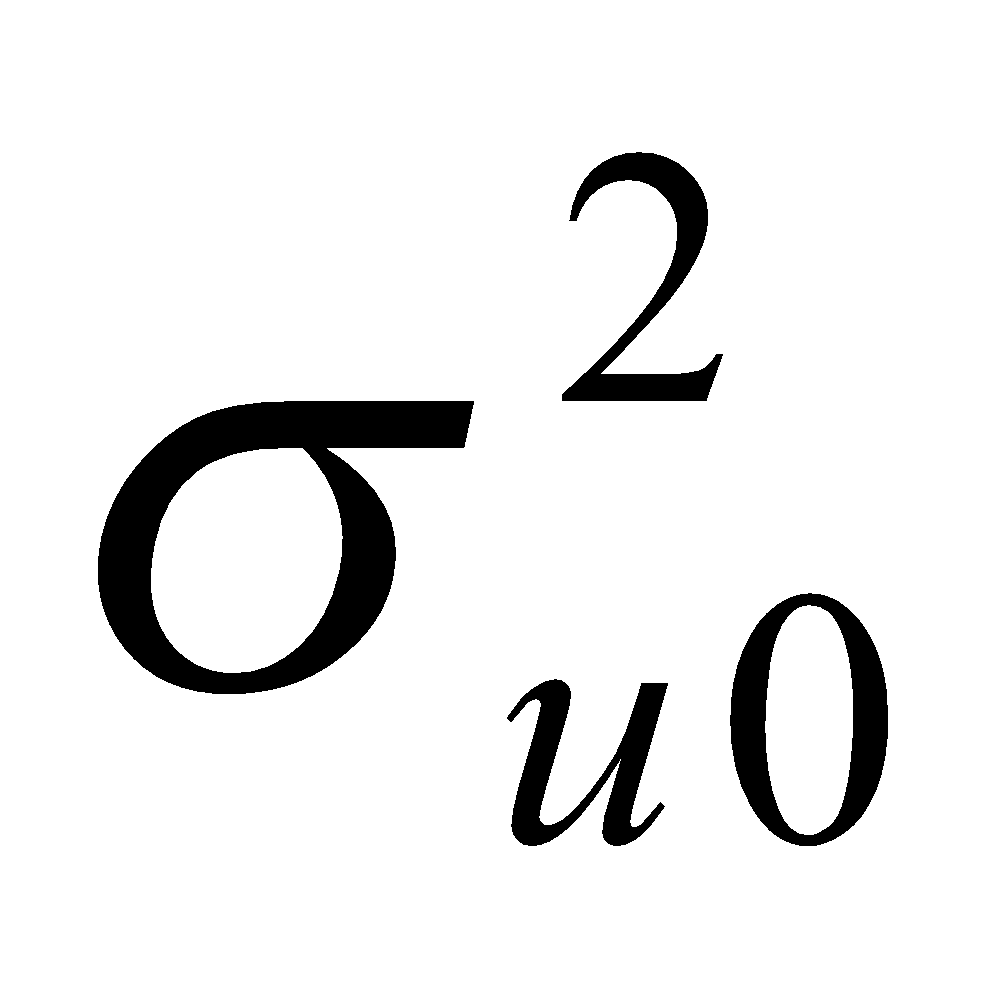_ | 3606.02***  (575.10) | 1235.46***  (172.49) | 1240.61***  (176.79) |
| _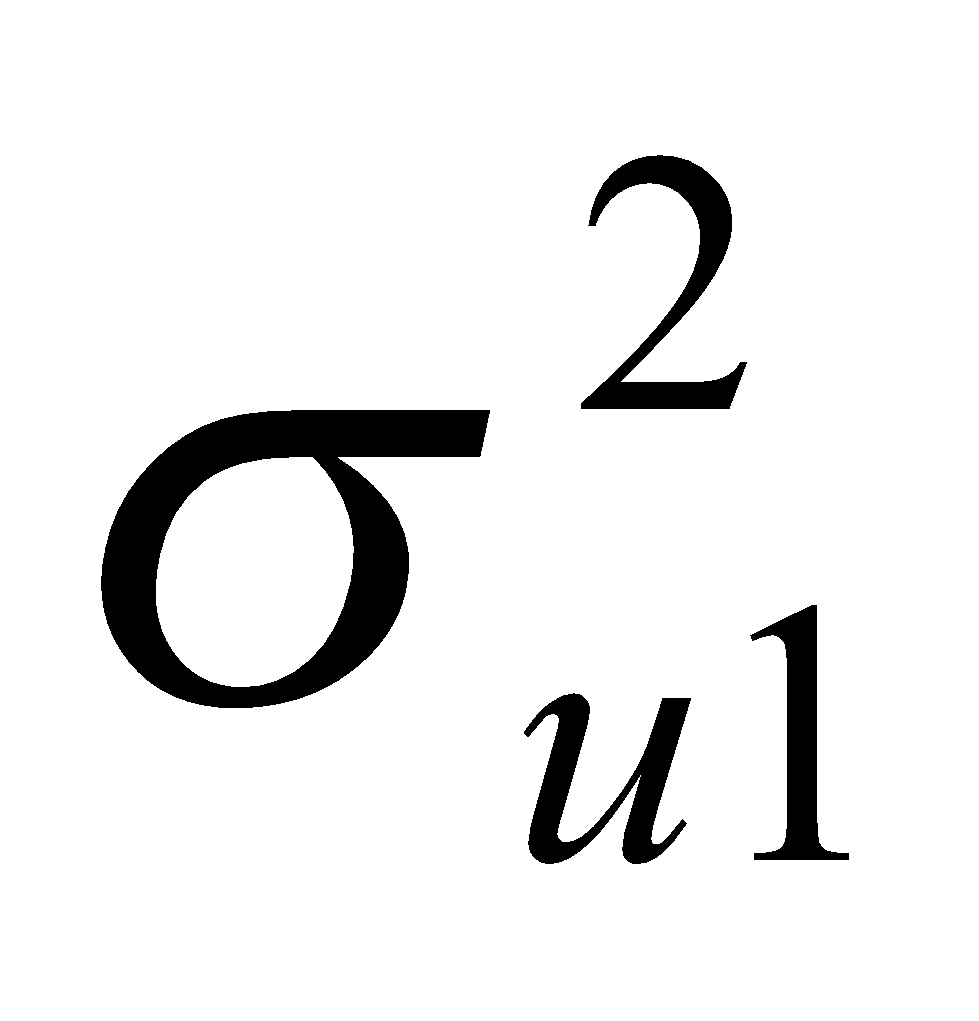_ |  |  | 187.29***  (48.20) |
| **ICC** | .46 |  |  |
| **AIC** | 11146642 | 11124181 | 11100362 |
| **BIC** | 11146664 | 11124307 | 11100502 |

*Note.* Standard errors in parentheses; Model (1) was the null model, model (2) was the model fitted with the fixed predictors, and model (3) was fitted with the fixed predictors and a random slope for relative belonging.

*p* < .10; **p* < .05; ***p* < .01; ****p* < .001

**Table A7**

*Two-level hierarchical models for reading outcome for girls-only sample*

|  | **Model 1** | **Model 2** | **Model 3** |
| --- | --- | --- | --- |
| **Fixed Effects** |  |  |  |
| Relative sense of belonging |  | 2.24  (1.55) | 3.10  (2.35) |
| Average school belonging |  | -12.00  (15.77) | -8.45  (15.33) |
| Age |  | -11.31*  (4.80) | -10.59*  (4.94) |
| Student ESCS |  | 10.06***  (1.54) | 9.92***  (1.56) |
| School ESCS |  | 54.41***  (8.42) | 54.93***  (8.32) |
| Girl proportion |  | 112.20**  (38.05) | 99.85**  (37.61) |
| School size |  | .00  (.00) | .00  (.00) |
| Class size |  | 1.26**  (.44) | 1.25**  (.42) |
| School type (private) |  | 1.06  (9.08) | 1.97  (9.35) |
| School location (small town) |  | .42  (13.21) | 1.32  (12.34) |
| School location (town) |  | 6.86  (14.03) | 5.06  (13.53) |
| School location (city) |  | -18.92  (16.49) | -21.13  (16.10) |
| School location (large city) |  | -4.26  (15.90) | -7.46  (15.37) |
| Student-teacher ratio |  | .30  (.93) | .32  (.90) |
| Intercept | 534.37***  (5.48) | 658.46***  (80.96) | 653.78***  (82.77) |
| **Random Effects** |  |  |  |
| _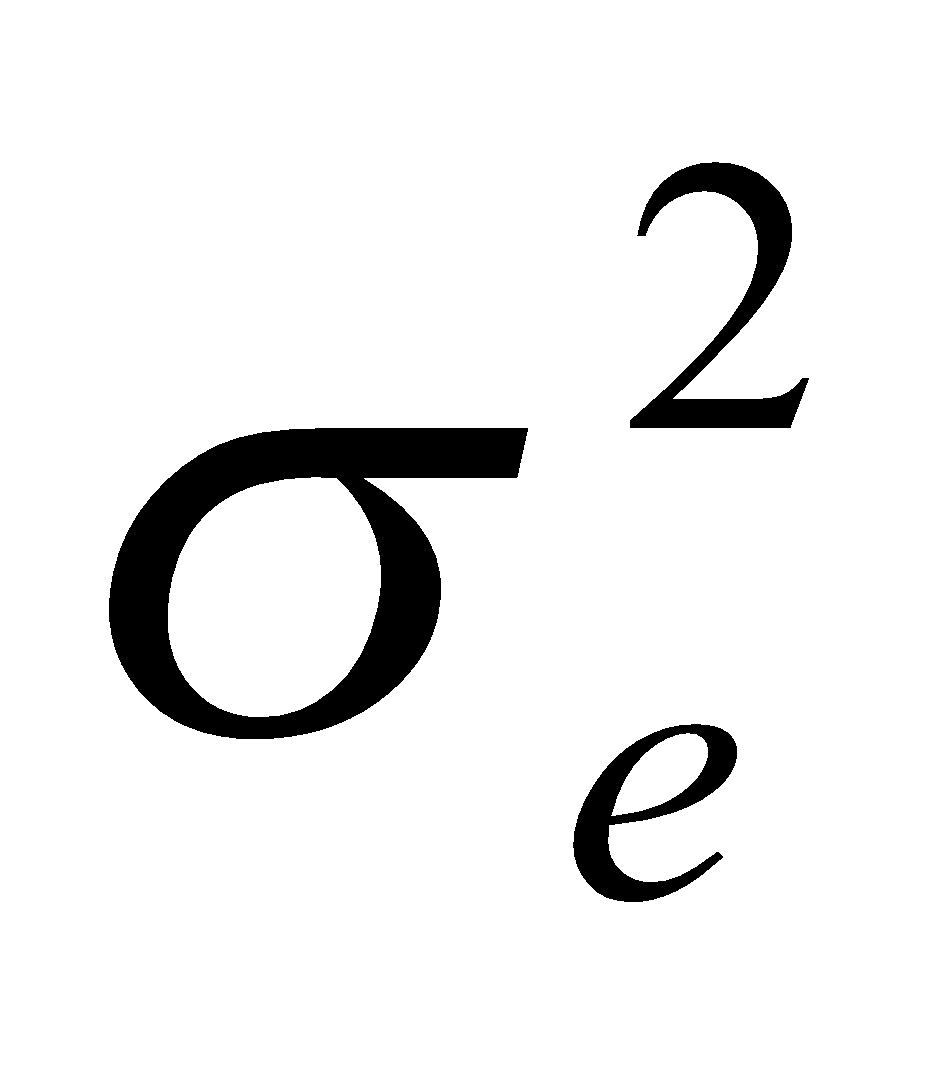_ | 3864.04***  (156.99) | 3776.90***  (149.93) | 3533.57***  (86.86) |
| _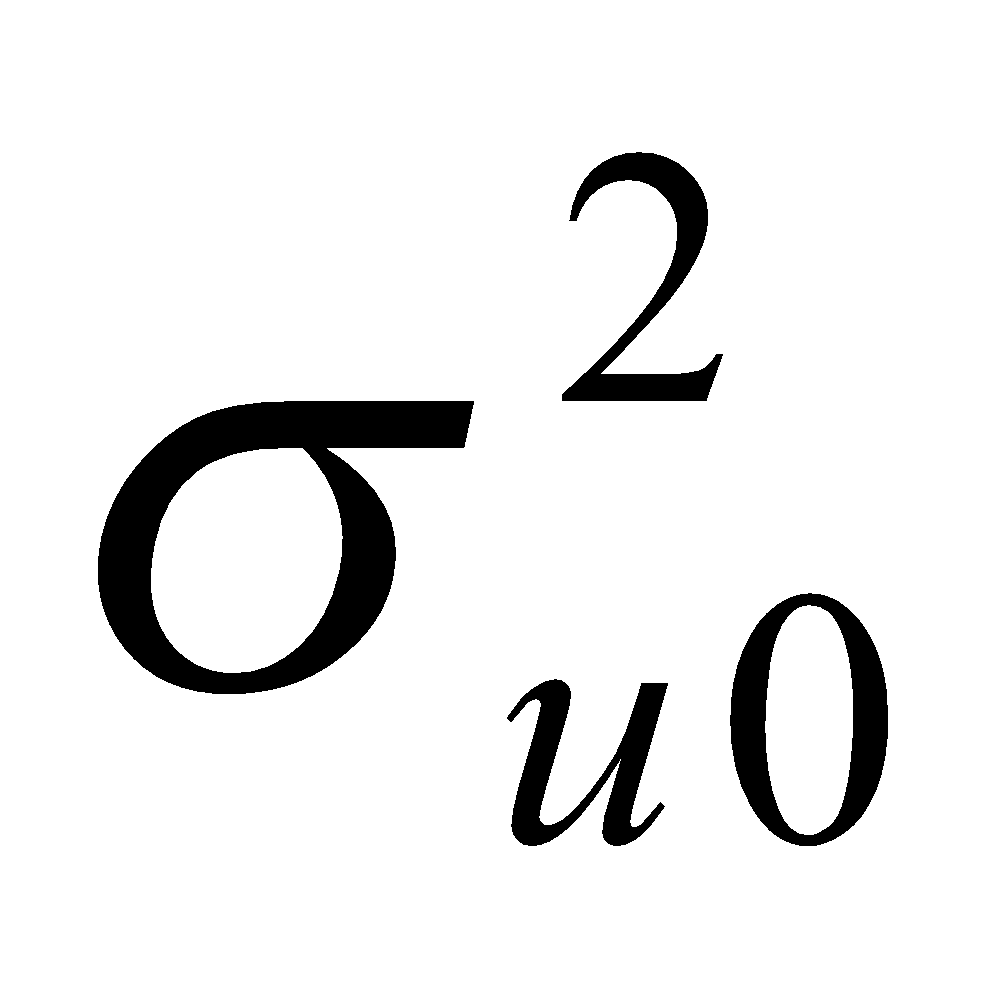_ | 3190.66***  (390.24) | 1055.77***  (141.87) | 1079.02***  (152.97) |
| _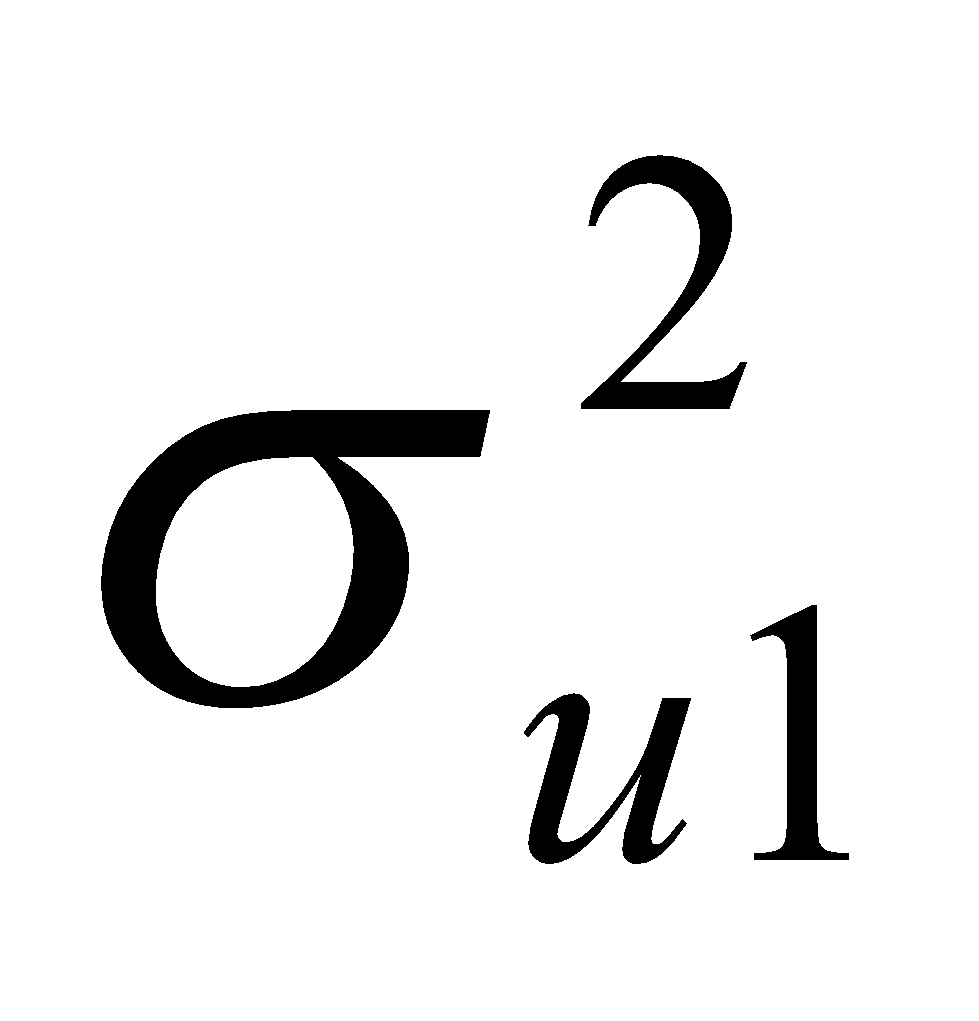_ |  |  | 326.18***  (84.07) |
| **ICC** | .45 |  |  |
| **AIC** | 5283709 | 5266601 | 5244910 |
| **BIC** | 5283729 | 5266714 | 5245037 |

*Notes:* Standard errors in parentheses; Model (1) was the null model, model (2) was the model fitted with the fixed predictors, and model (3) was fitted with the fixed predictors and a random slope for relative belonging.

*p* < .10; **p* < .05; ***p* < .01; ****p* < .001

**Table A8**

*Two-level hierarchical models for reading outcome for boys-only sample*

|  | **Model 1** | **Model 2** | **Model 3** |
| --- | --- | --- | --- |
| **Fixed Effects** |  |  |  |
| Relative sense of belonging |  | 6.29***  (1.65) | 8.54***  (2.55) |
| Average school belonging |  | 15.2  (21.1) | 14.6  (20.6) |
| Age |  | -.12*  (5.25) | -.12*  (5.48) |
| Student ESCS |  | 4.97**  (1.75) | 4.78**  (1.83) |
| School ESCS |  | 61.0***  (9.46) | 61.2***  (9.06) |
| Girl proportion |  | 133**  (43.1) | 124**  (46.4) |
| School size |  | .00  (.00) | .00  (.00) |
| Class size |  | 1.16*  (.58) | 1.19*  (.56) |
| School type (private) |  | 5.70  (10.2) | 1.57  (9.95) |
| School location (small town) |  | 8.49  (14.4) | 11.7  (14.4) |
| School location (town) |  | 17.0  (16.2) | 21.4  (16.1) |
| School location (city) |  | .69  (14.9) | 3.64  (14.8) |
| School location (large city) |  | -.68  (17.5) | -.16  (17.5) |
| Student-teacher ratio |  | .62  (1.31) | .79  (1.24) |
| Intercept | 521.40***  (6.62) | 649.00***  (85.6) | 644.00***  (89.3) |
| **Random Effects** |  |  |  |
| _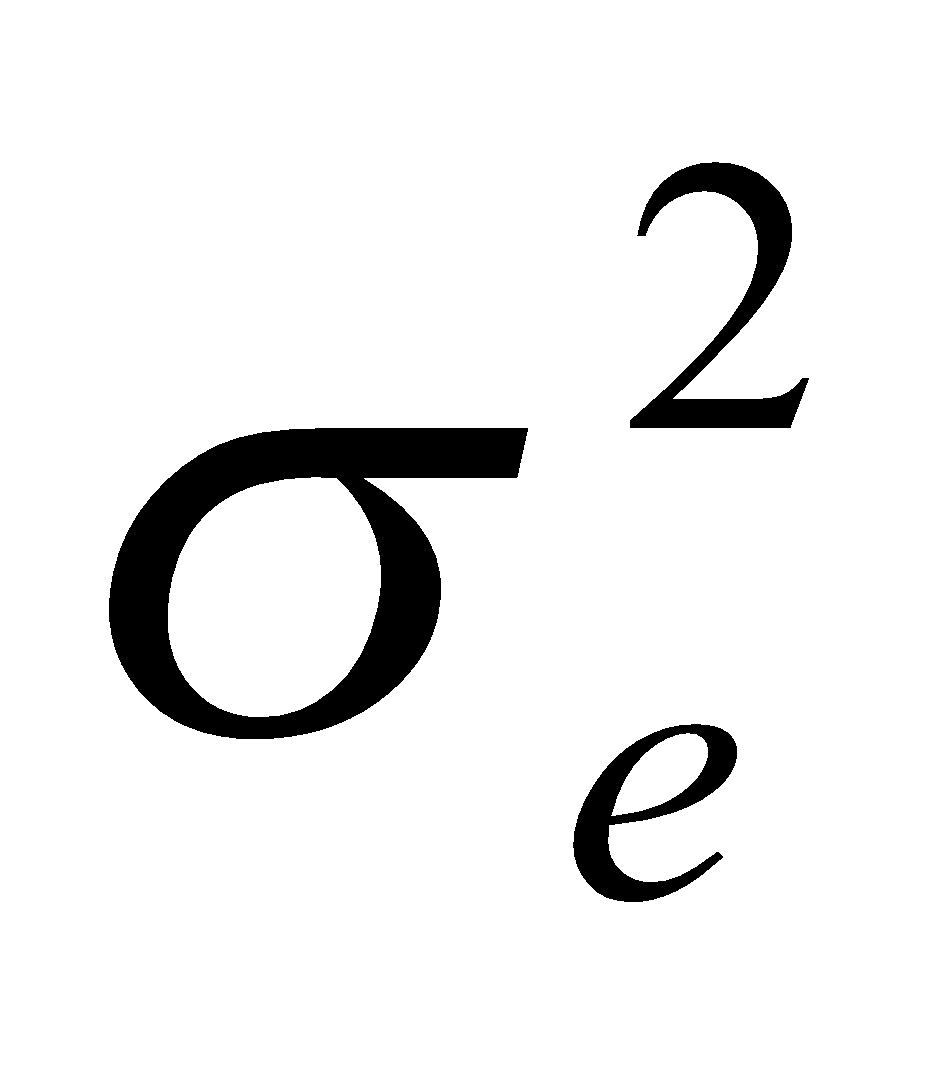_ | 4517.82***  (227.65) | 4448.39***  (215.90) | 4174.10***  (75.19) |
| _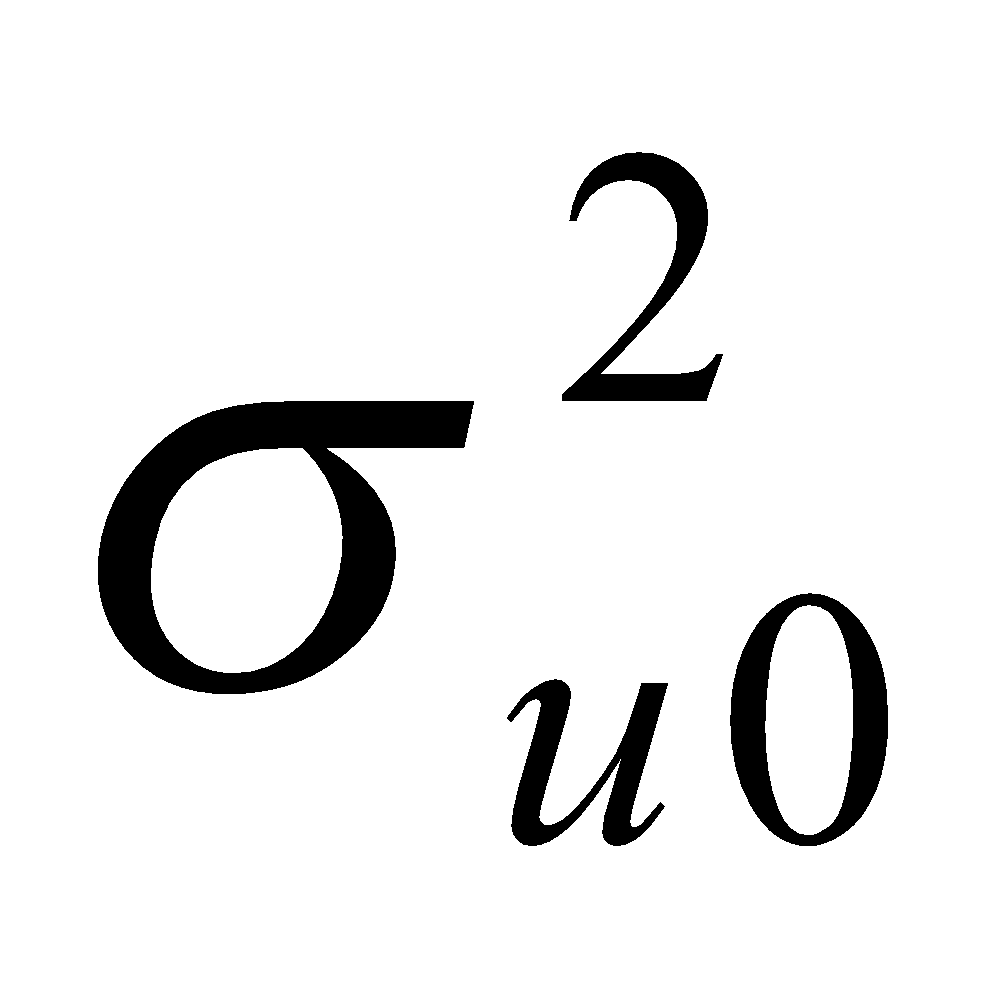_ | 3911.30***  (643.99) | 1368.79***  (202.38) | 1375.78***  (200.54) |
| _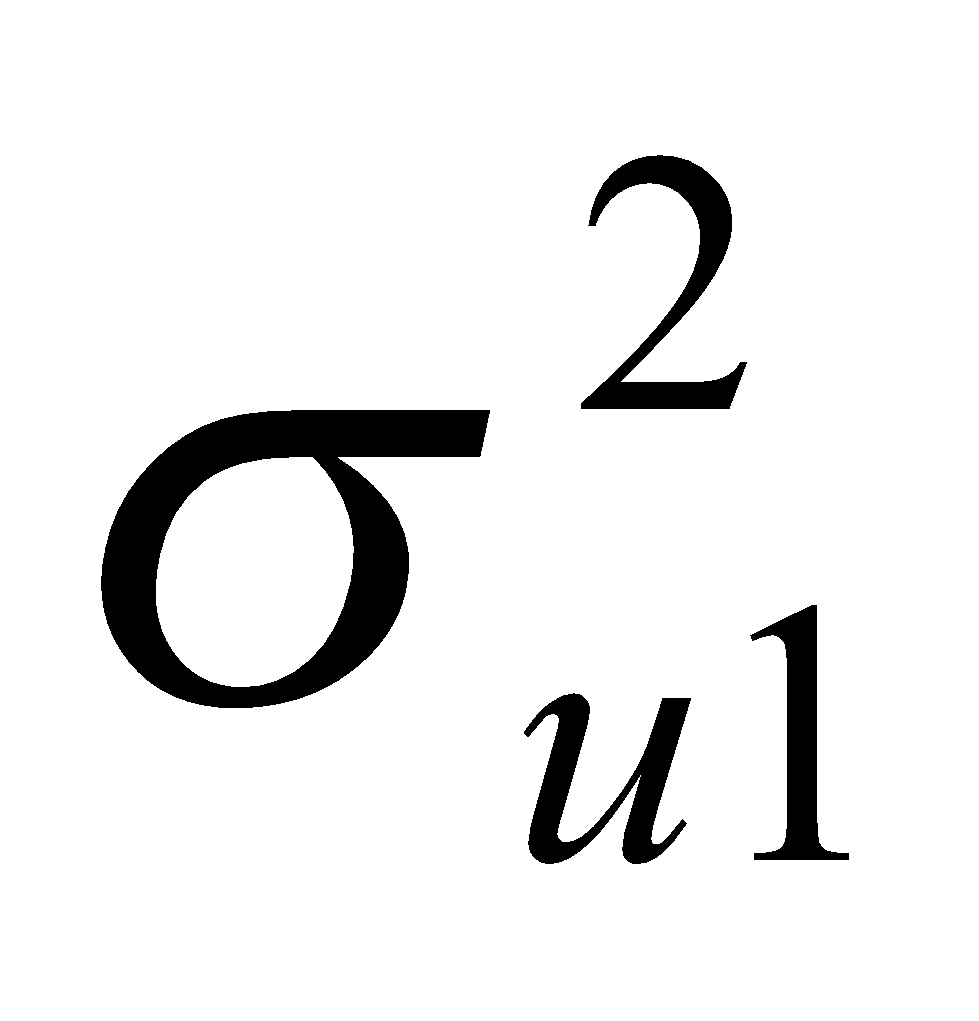_ |  |  | 346.40***  (82.92) |
| **ICC** | .46 |  |  |
| **AIC** | 5855663 | 5841511 | 5818692 |
| **BIC** | 5855684 | 5841626 | 5818820 |

*Notes:* Standard errors in parentheses; Model (1) was the null model, model (2) was the model fitted with the fixed predictors, and model (3) was fitted with the fixed predictors and a random slope for relative belonging.

*p* < .10; **p* < .05; ***p* < .01; ****p* < .001

**Table A9**

*Two-level hierarchical models for science outcome for the full sample*

|  | **Model 1** | **Model 2** | **Model 3** |
| --- | --- | --- | --- |
| **Fixed Effects** |  |  |  |
| Relative sense of belonging |  | 4.28**  (1.38) | 5.95***  (1.79) |
| Average school belonging |  | 5.06  (15.4) | 5.42  (14.0) |
| Age |  | -7.85  (4.80) | -7.60  (4.79) |
| Student ESCS |  | 5.14***  (1.28) | 5.10***  (1.28) |
| School ESCS |  | 57.5***  (8.16) | 56.2***  (7.49) |
| Girl proportion |  | 125**  (40.7) | 111**  (40.9) |
| School size |  | .00  (.00) | .00  (.00) |
| Class size |  | 1.03*  (.51) | 1.03*  (.47) |
| School type (private) |  | 2.40  (8.66) | -.18  (8.76) |
| School location (small town) |  | 2.94  (13.1) | 5.62  (11.7) |
| School location (town) |  | 10.9  (13.9) | 13.4  (12.8) |
| School location (city) |  | -12.4  (14.2) | -9.89  (13.0) |
| School location (large city) |  | -8.03  (15.5) | -8.13  (14.4) |
| Student-teacher ratio |  | .87  (1.17) | .94  (1.03) |
| Intercept | 563.88***  (5.75) | 630.00***  (79.9) | 630***  (79.0) |
| **Random Effects** |  |  |  |
| _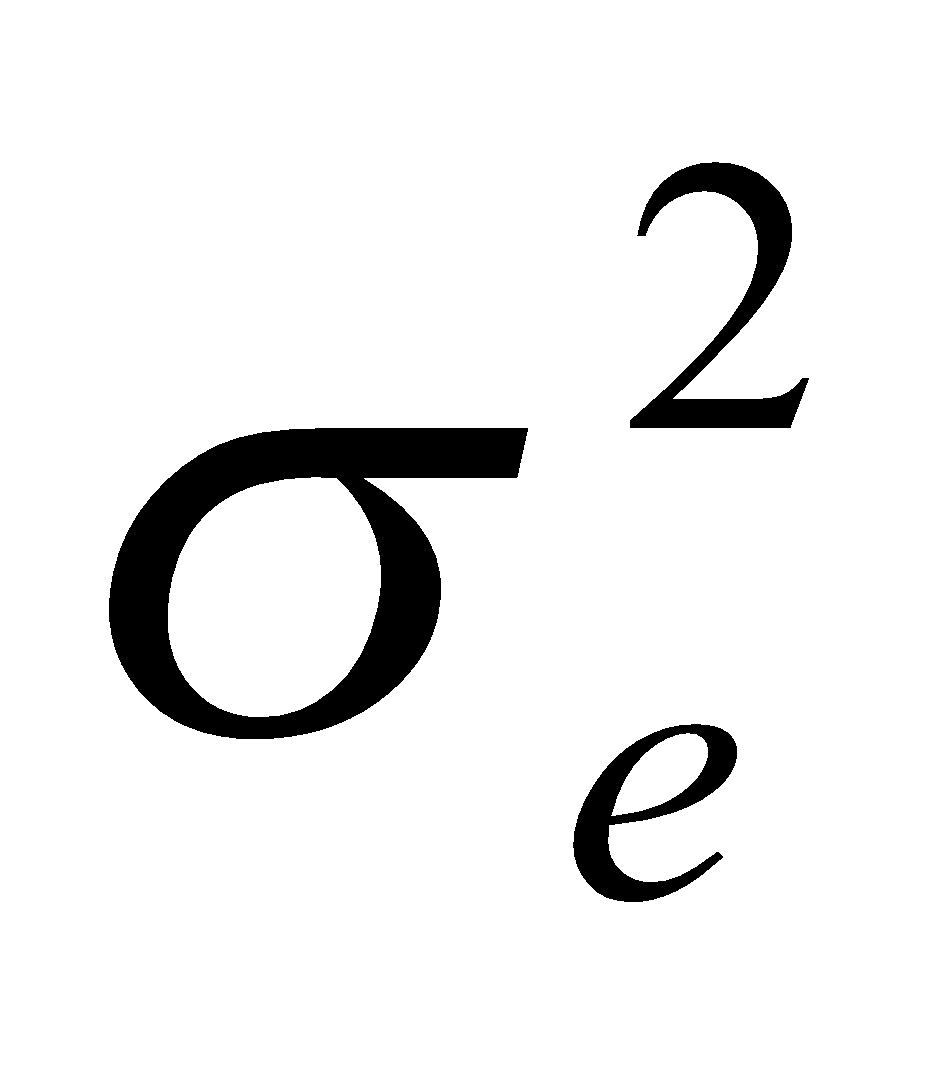_ | 3912.60***  (145.44) | 3867.64***  (141.66) | 3762.76***  (67.18) |
| _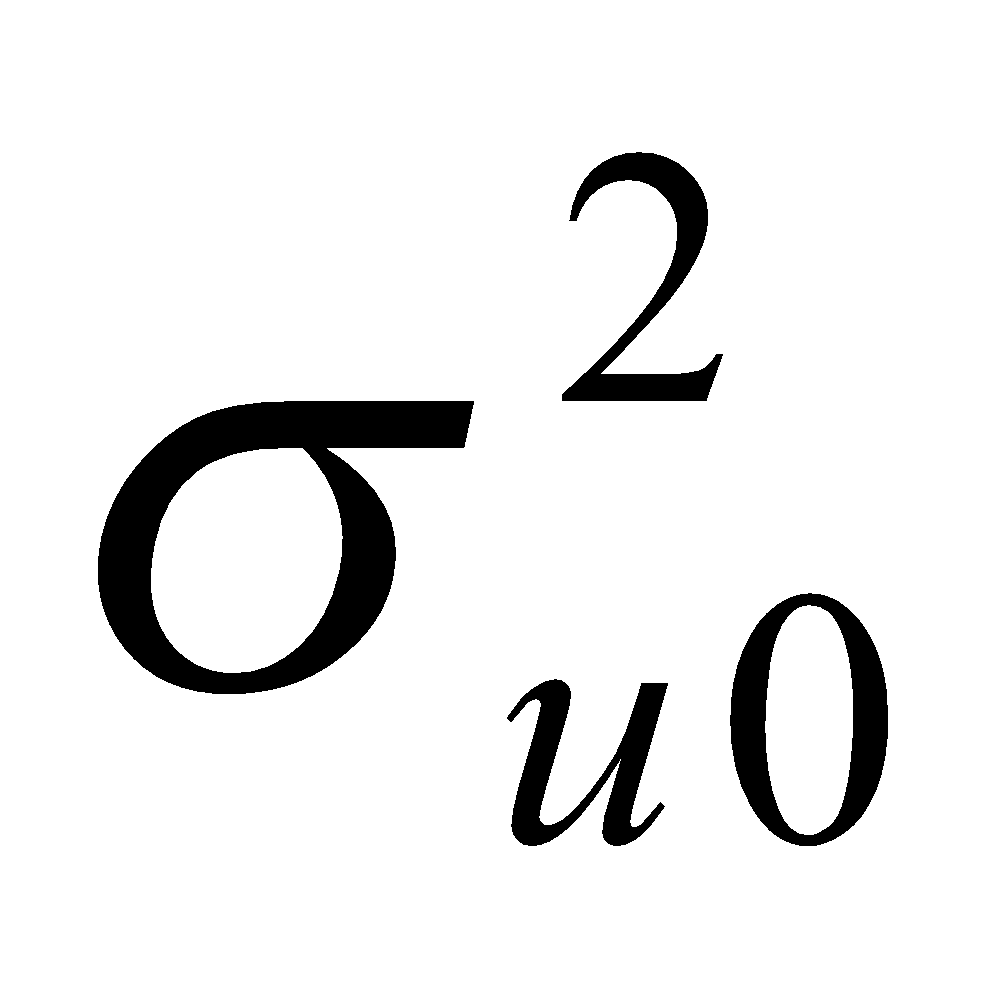_ | 3212.46***  (505.31) | 1123.12***  (149.07) | 1125.25***  (150.92) |
| _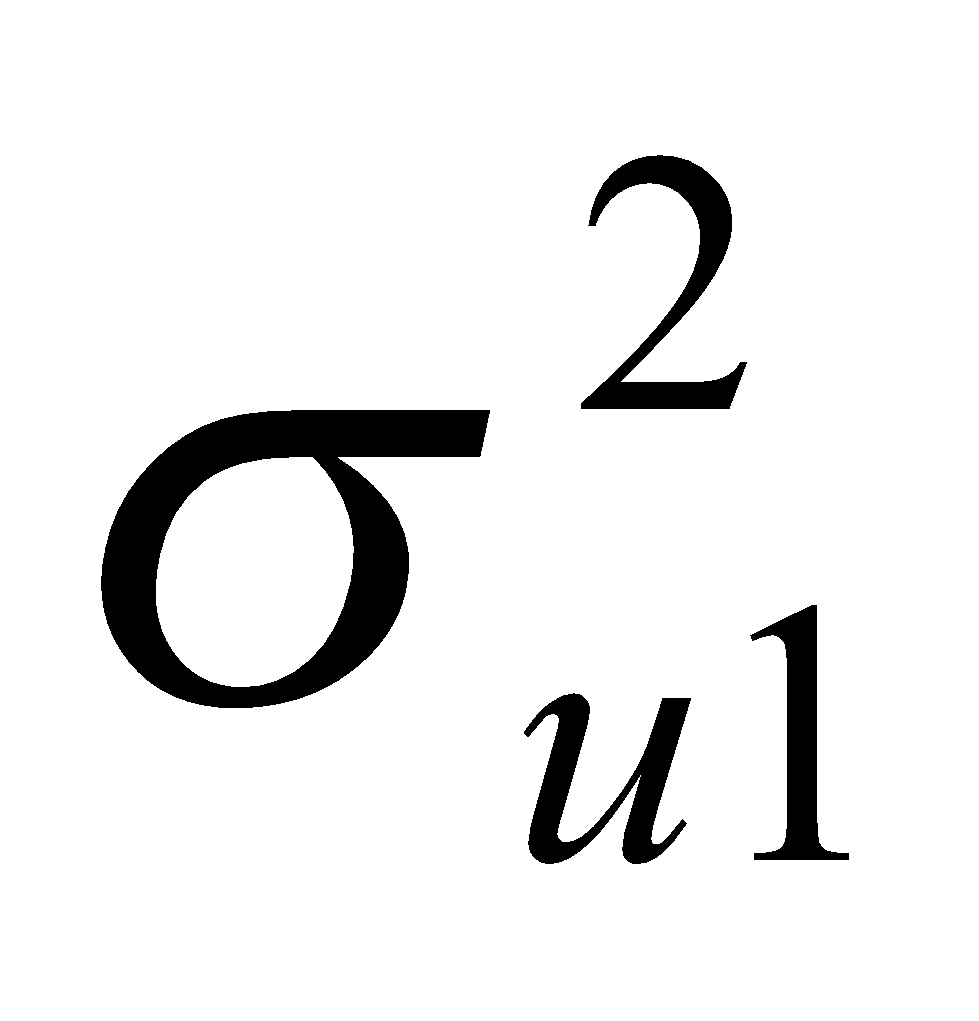_ |  |  | 135.22  (36.62) |
| **ICC** | .45 |  |  |
| **AIC** | 11051717 | 11033926 | 11015626 |
| **BIC** | 11051739 | 11034052 | 11015767 |

*Note.* Standard errors in parentheses; Model (1) was the null model, model (2) was the model fitted with the fixed predictors, and model (3) was fitted with the fixed predictors and a random slope for relative belonging.

*p* < .10; **p* < .05; ***p* < .01; ****p* < .001

**Table A10**

*Two-level hierarchical models for science outcome for girls-only sample*

|  | **Model 1** | **Model 2** | **Model 3** |
| --- | --- | --- | --- |
| **Fixed Effects** |  |  |  |
| Relative sense of belonging |  | 2.66  (1.57) | 4.19  (2.53) |
| Average school belonging |  | -8.73  (14.49) | -5.78  (13.99) |
| Age |  | -9.90  (5.54) | -9.49  (5.61) |
| Student ESCS |  | 6.80***  (1.48) | 6.78***  (1.52) |
| School ESCS |  | 53.09***  (7.98) | 53.40***  (7.62) |
| Girl proportion |  | 123.75**  (40.14) | 106.18**  (39.34) |
| School size |  | .00  (.00) | .00  (.00) |
| Class size |  | 1.11*  (.46) | 1.09*  (.43) |
| School type (private) |  | 2.65  (8.66) | 3.63  (8.82) |
| School location (small town) |  | .86  (12.82) | 2.59  (11.98) |
| School location (town) |  | 7.10  (13.57) | 6.26  (12.76) |
| School location (city) |  | -17.97  (15.79) | -19.14  (15.16) |
| School location (large city) |  | -5.96  (15.37) | -8.75  (14.49) |
| Student-teacher ratio |  | .44  (.96) | .52  (.88) |
| Intercept | 557.89***  (5.19) | 655.53***  (91.87) | 657.52***  (92.46) |
| **Random Effects** |  |  |  |
| _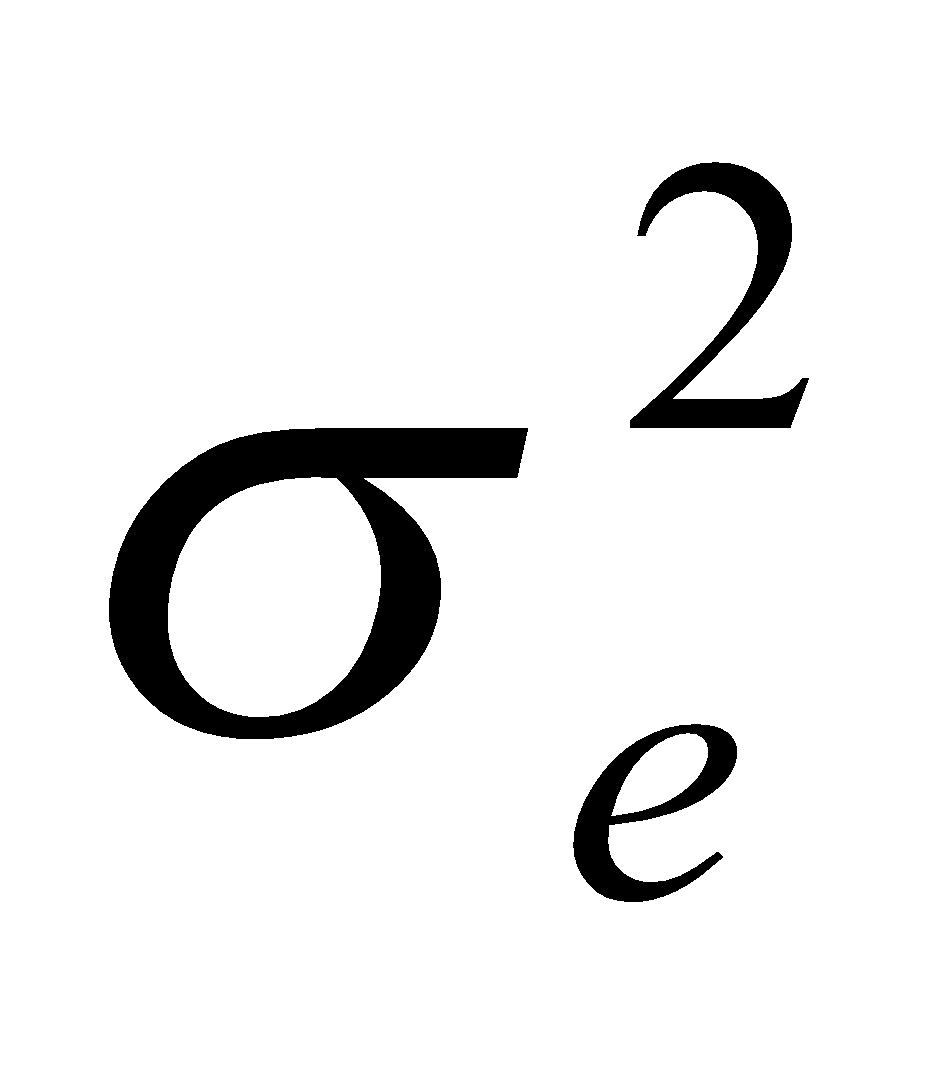_ | 3436.09***  (151.70) | 3386.16***  (145.88) | 3177.59***  (100.17) |
| _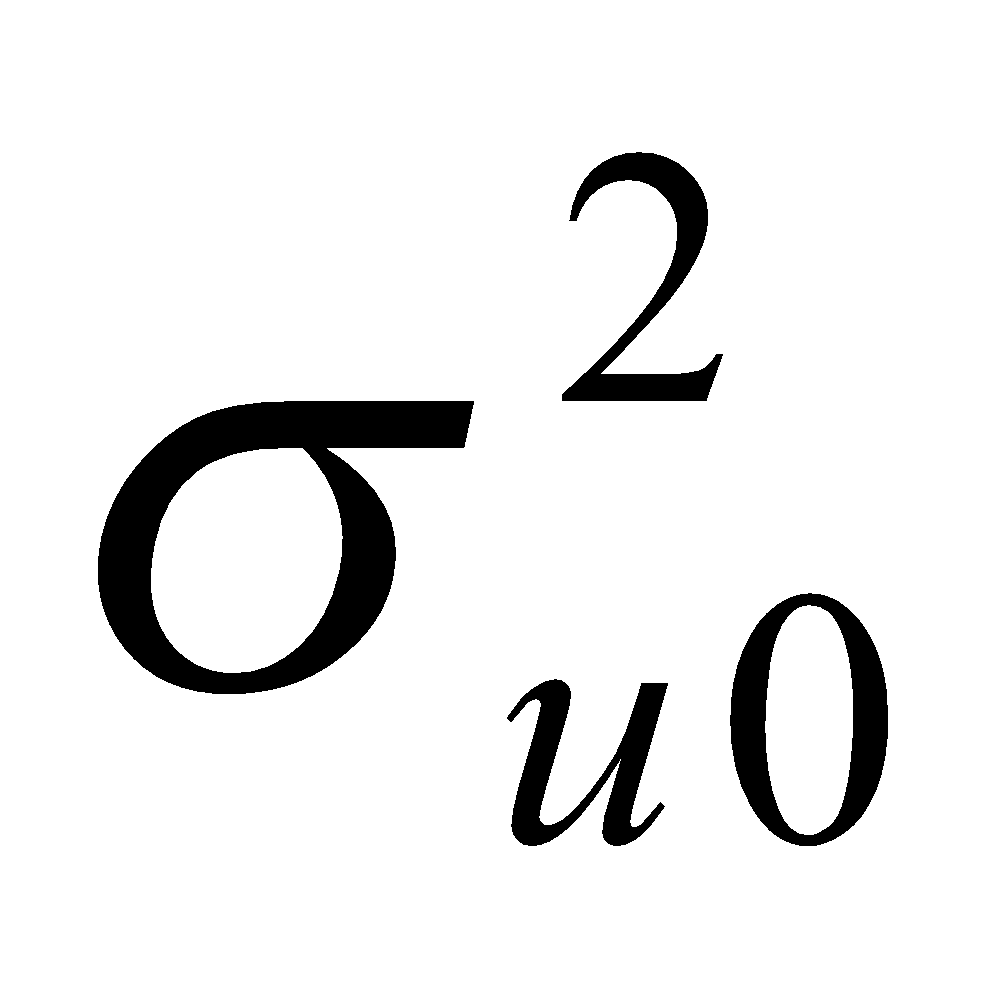_ | 2863.29***  (372.60) | 1043.08***  (139.57) | 1071.82***  (151.87) |
| _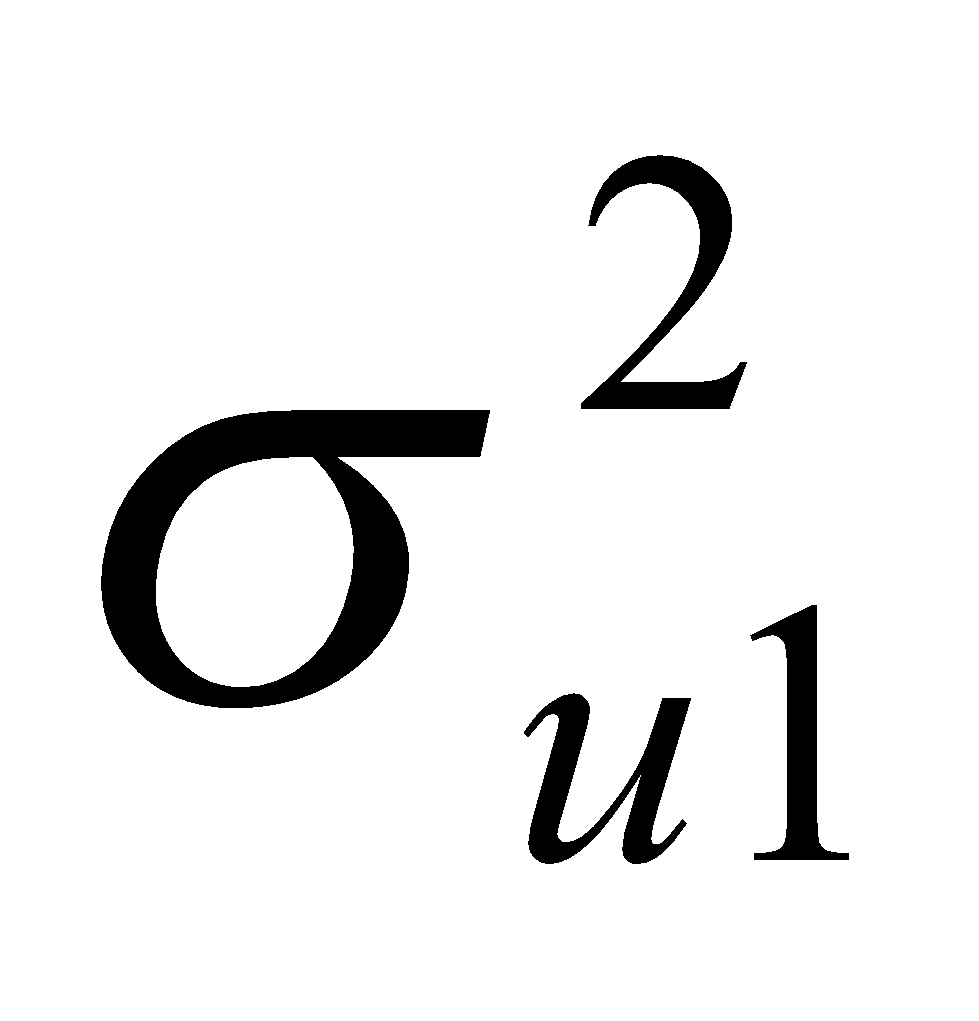_ |  |  | 294.23***  (83.05) |
| **ICC** | .45 |  |  |
| **AIC** | 5228052 | 5215319 | 5194960 |
| **BIC** | 5228072 | 5215432 | 5195087 |

*Notes:* Standard errors in parentheses; Model (1) was the null model, Model (2) was the model fitted with the fixed predictors, and Model (3) was fitted with the fixed predictors and a random slope for relative belonging.

*p* < .10; **p* < .05; ***p* < .01; ****p* < .001

**Table A11**

*Two-level hierarchical models for science outcome for the boys-only sample*

|  | **Model 1** | **Model 2** | **Model 3** |
| --- | --- | --- | --- |
| **Fixed Effects** |  |  |  |
| Relative sense of belonging |  | 5.03**  (1.85) | 6.71**  (2.42) |
| Average school belonging |  | 18.1  (20.1) | 17.08  (19.76) |
| Age |  | -8.15  (5.98) | -7.94  (6.06) |
| Student ESCS |  | 3.72*  (1.66) | 3.67*  (1.71) |
| School ESCS |  | 59.70***  (8.77) | 58.98***  (8.50) |
| Girl proportion |  | 150***  (43.2) | 142.26**  (45.02) |
| School size |  | .00  (.00) | -.00  (.00) |
| Class size |  | 1.11*  (.56) | 1.13*  (.55) |
| School type (private) |  | 3.65  (9.19) | 1.29  (9.26) |
| School location (small town) |  | 2.55  (14.6) | 3.94  (13.94) |
| School location (town) |  | 16.40  (15.4) | 19.23  (15.04) |
| School location (city) |  | -4.46  (14.9) | -2.04  (14.34) |
| School location (large city) |  | -9.08  (16.9) | -7.97  (16.45) |
| Student-teacher ratio |  | .91  (1.20) | .96  (1.16) |
| Intercept | 570.37***  (6.25) | 627.00***  (96.8) | 624.36***  (98.21) |
| **Random Effects** |  |  |  |
| _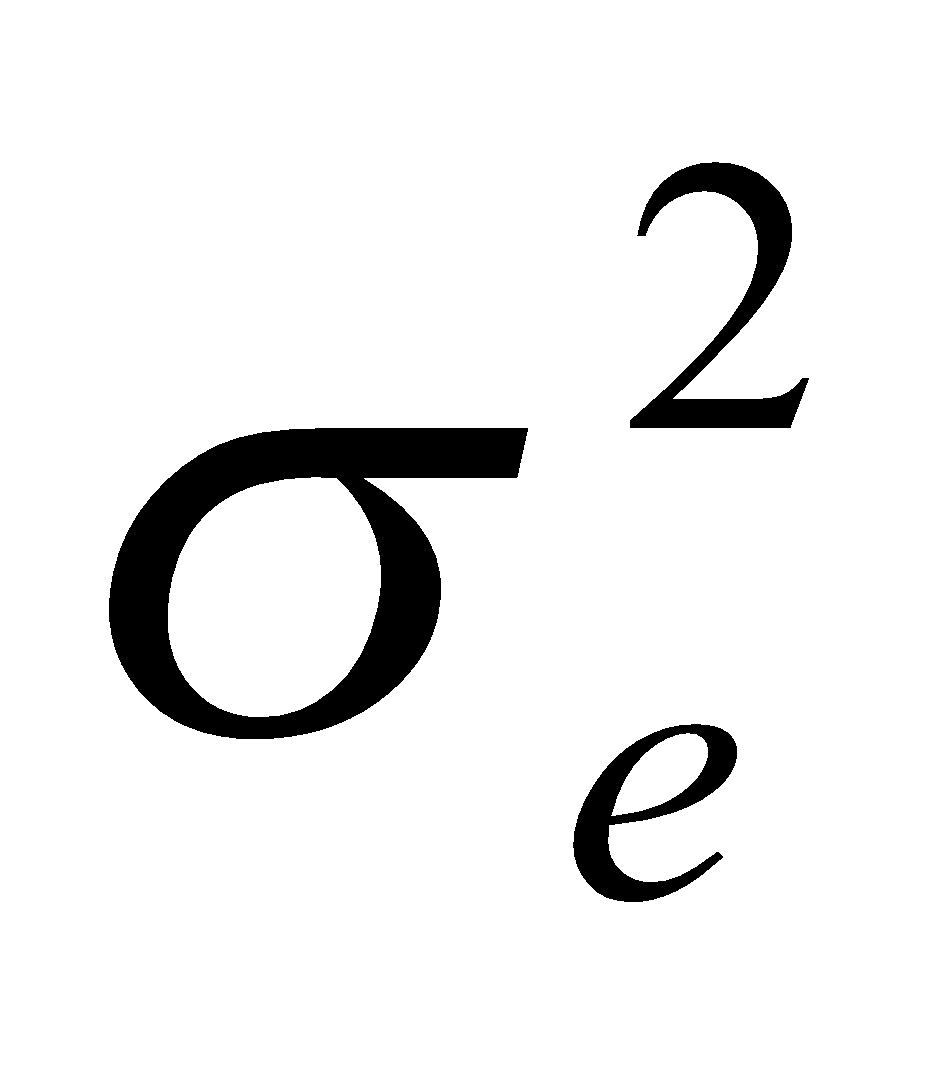_ | 4036.64***  (197.30) | 3993.65***  (191.26) | 3771.28***  (85.84) |
| _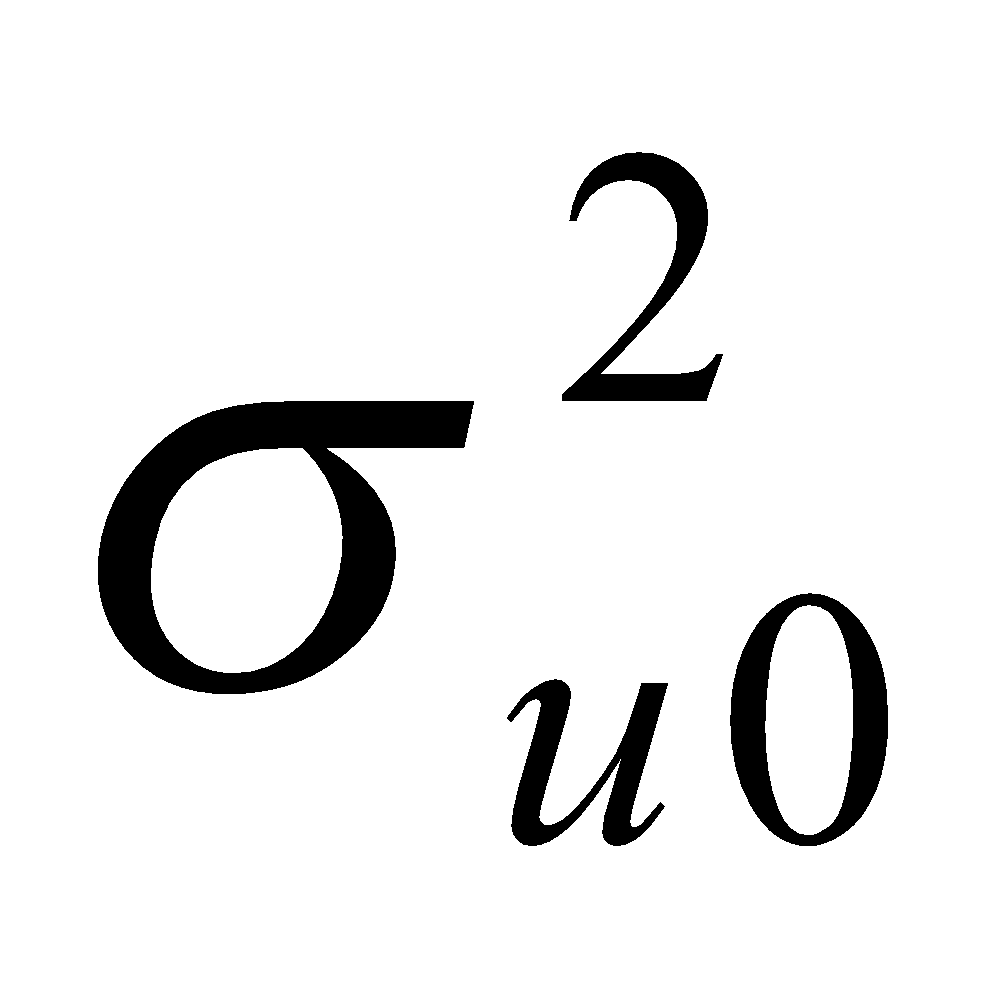_ | 3584.63***  (602.50) | 1258.80***  (179.46) | 1264.68***  (176.76) |
| _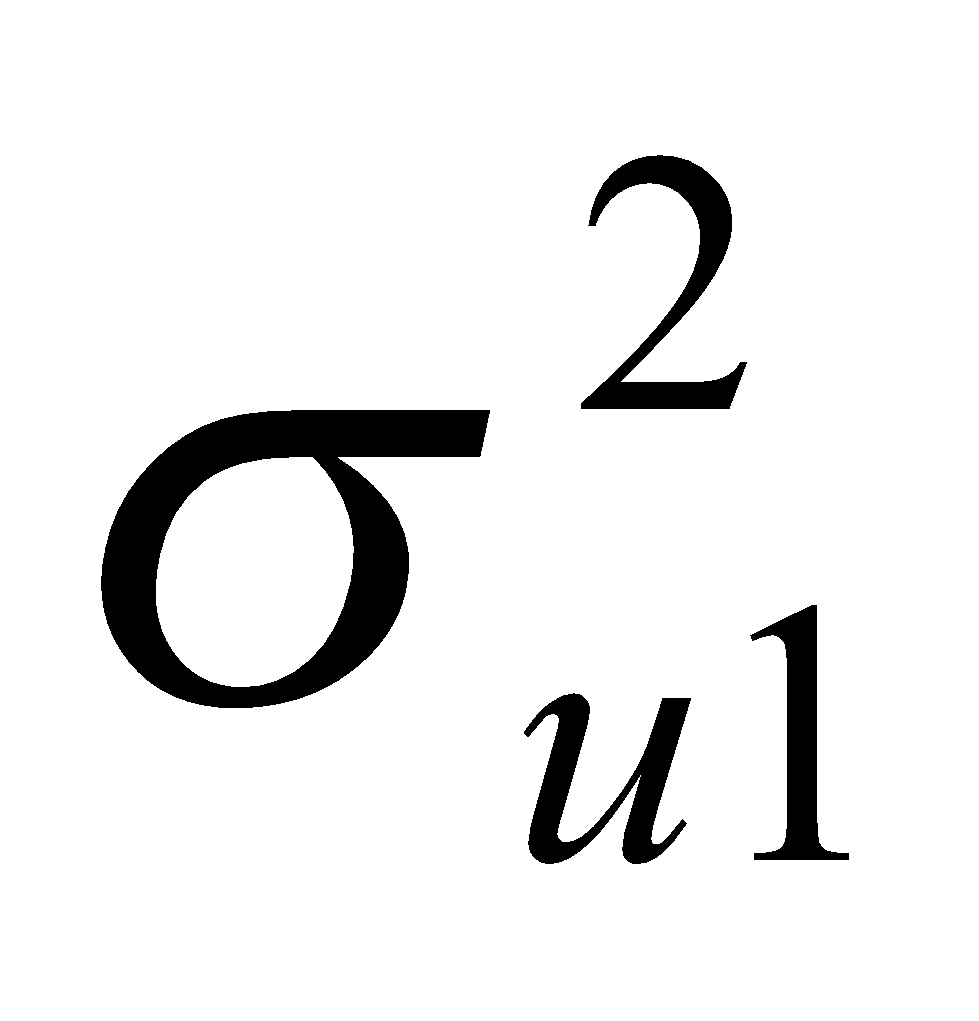_ |  |  | 284.80***  (77.13) |
| **ICC** | .47 |  |  |
| **AIC** | 5797405 | 5785715 | 5765769 |
| **BIC** | 5797426 | 5785830 | 5765897 |

*Notes:* Standard errors in parentheses; Model (1) was the null model, model (2) was the model fitted with the fixed predictors, and model (3) was fitted with the fixed predictors and a random slope for relative belonging.

*p* < .10; **p* < .05; ***p* < .01; ****p* < .001
