# Supplementary material for: Relative Sense of Belonging and the Academic Achievement of Chinese Adolescents
Source: J Youth Adolesc. 2025 Nov 13;55(4):918–32. doi: 10.1007/s10964-025-02287-5 (PMC13076513; doi:10.1007/s10964-025-02287-5)
Supplement: Supplementary file 2 — Supplementary Material 2 [file 10964_2025_2287_MOESM2_ESM.docx]

***Overall Sample***

Level 1：

$Y_{ij}$= $ꞵ_{oj}$+ $ꞵ_{1j}{relative sense of belonging}_{ij}$ + $ꞵ_{2j}student {ESCS}_{ij}$ + $ꞵ_{3j}{age}_{ij}$ + $e_{ij}$

Level 2:

$ꞵ_{oj}$= $\gamma_{00}$ + $\gamma_{01}{average school belonging}_{ij}$ + $\gamma_{02}{school ESCS}_{ij}$ + $\gamma_{03}{girl proportion}_{ij}$ + $\gamma_{04}{school size}_{ij}$ + $\gamma_{05}{school type}_{ij}$ + $\gamma_{06}{student teacher ratio}_{ij}$ + $\gamma_{07}{class size}_{ij}$ + $\gamma_{08}{school location}_{ij}$ + $\mu_{0j}$

$ꞵ_{1j}$ = $\gamma_{10}$ + $\mu_{1j}$

$ꞵ_{pj}$ = $\gamma_{p0}$ , for *p* = 2, 3,…,8
